# An Oral Antiviral Combination Against Severe COVID‐19 Enabled by the Synergistic Mechanisms of Action of Auranofin and Remdesivir

**DOI:** 10.1002/advs.78336

**Published:** 2026-10-22

**Authors:** Oyahida Khatun, Rohan Narayan, Santhosh Kambaiah Nagaraj, Rishad Shiraz, Abinaya Kaliappan, Sumandeep Kaur, Ankur Singh, Siva Shanmugam Selvaraj, Gudepalya Renukaiah Rudramurthy, Chakenahalli Naveenkumar, Shridhar Narayanan, Radha Krishan Shandil, Shailly Tomar, Shashank Tripathi

**Affiliations:** ^1^ Emerging Viral Pathogens Laboratory Infosys Wing Centre for Infectious Disease Research Indian Institute of Science Bengaluru India; ^2^ Department of Microbiology and Cell Biology Division of Biological Sciences Indian Institute of Science Bengaluru India; ^3^ Molecular Virology Lab Department of Bioscience and Bioengineering Indian Institute of Technology Roorkee Roorkee Uttarakhand India; ^4^ Foundation For Neglected Disease Research KIADB Industrial Area Bengaluru India

**Keywords:** antiviral, auranofin, Remdesivir, SARS‐CoV‐2, synergy, virus entry

## Abstract

Auranofin, an FDA‐approved oral anti‐rheumatic drug, was previously shown by our group to restrict Severe Acute Respiratory Syndrome Coronavirus‐2 (SARS‐CoV‐2) replication and pathology in preclinical models. Here, we systematically characterized its mechanism of action (MOA) through biochemical drug‐protein interactions, cell‐based mechanistic assays, and preclinical efficacy studies. Auranofin directly binds and inhibits SARS‐CoV‐2 MPro while simultaneously targeting multiple stages of the viral life cycle, disrupting endosomal trafficking, suppressing Spike S1/S2 proteolytic processing, impairing syncytia formation, and attenuating NF‐κB‐driven proinflammatory signaling. In cell culture, Auranofin demonstrated robust synergy with Remdesivir but competed with the MPro inhibitor Nirmatrelvir, informing rational combination design. In mice infected with the SARS‐CoV‐2 Delta variant, the oral combination of Auranofin and the Remdesivir prodrug GS‐621763 outperformed monotherapy, markedly reducing viral replication, inflammatory cytokine responses, and lung pathology, and improving clinical outcomes. These findings establish a multi‐target antiviral MOA of Auranofin and provide a mechanistic rationale for an all‐oral antiviral combination regimen against COVID‐19.

## Graphical abstract

FDA‐approved anti‐inflammatory drug Auranofin inhibits SARS‐CoV‐2 replication by inhibiting viral Main protease activity and Spike processing required for viral entry. It shows strong synergy with nucleoside analogue Remdesivir, due to non‐overlapping mechanisms. In a murine model of severe SARS‐CoV‐2 challenge, the oral Auranofin–Remdesivir prodrug combination provides robust protection, paving the way for an oral combination therapy for severe COVID‐19.

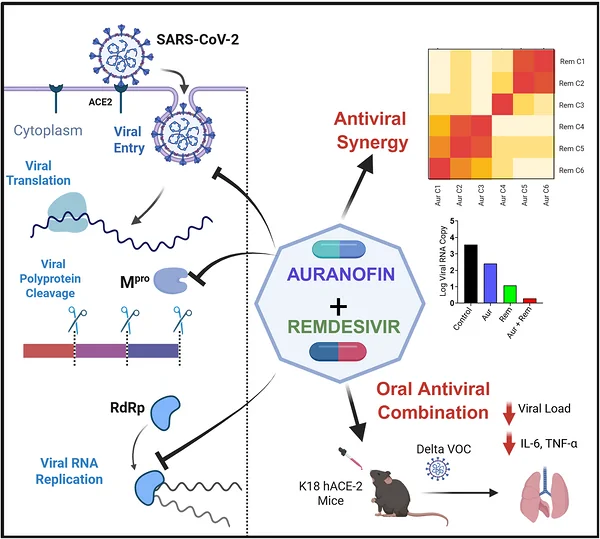

## 1 Introduction

COVID‐19, caused by Severe Acute Respiratory Syndrome Coronavirus‐2 (SARS‐CoV‐2), an enveloped, single‐stranded, positive‐sense RNA virus, has been one of the most devastating infectious disease crises in modern history [1, 2]. Since the first cases were reported in December 2019, the COVID‐19 pandemic has claimed over 7.1 million lives globally as of May 2026 [3]. Mortality was disproportionately concentrated among older adults and individuals with comorbidities, driven by a combination of direct viral tissue injury and dysregulated host immune responses [4, 5]. The subsequent development and deployment of vaccines and antiviral therapeutics substantially reduced COVID‐19‐associated morbidity and mortality worldwide [6]. Nevertheless, SARS‐CoV‐2 continues to evolve and circulate in the human population, causing recurrent outbreaks, underscoring the ongoing need for effective therapeutic strategies [7]. Antivirals represent the critical first line of defense against emerging pathogens, particularly in the absence of vaccines, and play an indispensable complementary role by reducing viral burden and limiting transmission even when vaccines are available [8]. Antiviral agents broadly fall into two mechanistic categories: Direct‐Acting Antivirals (DAAs) and Host‐Directed Antivirals (HDAs) [9]. For SARS‐CoV‐2, DAAs include anti‐spike monoclonal antibodies targeting viral entry [10], protease inhibitors such as Nirmatrelvir [11], and RNA‐dependent RNA polymerase (RdRp) inhibitors such as Remdesivir that disrupt viral genome replication and transcription [12, 13]. A key liability of DAAs, particularly those targeting viral entry and protease, is their susceptibility to resistance mutations [13, 14].

Although nucleoside analogue RdRp inhibitors are comparatively more resistant to such escape [15], they have limitations in oral bioavailability and safety, especially with Remdesivir [16, 17]. HDAs, by contrast, target host factors co‐opted during the viral lifecycle or modulate the pathological inflammatory response [18, 19, 20]. Notable examples in the context of COVID‐19 include tocilizumab, an immunosuppressive anti‐IL‐6 receptor monoclonal antibody [21, 22], and baricitinib, a JAK inhibitor with anti‐inflammatory properties [23]. Hydroxychloroquine, though investigated as an HDA, demonstrated no proven efficacy in controlled clinical trials [24]. The limitations of DAA or HDA monotherapy underscore the need for combination strategies that achieve synergistic antiviral and immunomodulatory effects through rational, mechanistically informed design [25, 26]. Despite the substantial efforts of the scientific community to develop effective therapies against coronaviruses, there remains no widely accepted consensus on an effective combination therapy [27, 28].

Auranofin, an FDA‐approved gold‐containing compound used to treat rheumatoid arthritis, has emerged as a compelling HDA candidate to address this gap [29]. Our previous work demonstrated that Auranofin exhibits antiviral activity against SARS‐CoV‐2 in cell culture and animal models, with its in vivo efficacy associated with suppression of the pro‐inflammatory cytokine IL‐6 [30]. These findings were subsequently corroborated by independent studies confirming Auranofin's antiviral properties and linking its activity to modulation of cellular redox and inflammatory signaling [31, 32], although its direct interactions with viral targets remained incompletely characterized. In the present study, we systematically define the mechanism of action of Auranofin against SARS‐CoV‐2. We demonstrate that Auranofin impairs viral entry and syncytia formation by interfering with spike protein processing and by directly binding to and inhibiting MPro. Notably, Auranofin does not affect viral RdRp activity, establishing a mechanistic basis for combination with nucleoside analogues, such as Remdesivir. Accordingly, Auranofin displayed synergistic antiviral activity with Remdesivir in vitro, motivating evaluation of an all‐oral combination regimen in vivo. This combination demonstrated superior antiviral efficacy compared with either agent alone, as evidenced by improved clinical outcomes, reduced viral load, and attenuated inflammatory marker expression. Together, these findings provide preclinical validation for a mechanistically informed, all‐oral Auranofin–Remdesivir combination therapy against COVID‐19.

## 2 Results

### 2.1 Auranofin Impairs SARS‐CoV‐2 Entry Into Host Cells and Impedes Endosomal Trafficking of Virus Particles

To understand the mode of action of Auranofin against SARS‐CoV‐2, we initially tested its effect on virus entry using luciferase‐expressing non‐replicating SARS‐CoV‐2 spike pseudotyped virus particles in HEK293 cells expressing the human ACE2 receptor (HEK293‐ACE2) cells (Figure 1A) [33]. Treatment with 1 µM Auranofin reduced entry of pseudotyped particles by 3‐fold as measured by luciferase activity 48 h post infection (hpi) (Figure 1B). We then validated this finding in an entry assay where HEK293‐ACE2 were pre‐treated with 1 µM Auranofin for 3 h and infected with 10 multiplicity of infection (MOI) SARS‐CoV‐2 (Hong Kong/VM20001061/2020 isolate) (hereby referred as SARS‐CoV‐2) in the presence of the drug (Figure 1C). After 3 h of infection, cell lysates were collected and analyzed by Western blot. Auranofin treatment significantly reduced SARS‐CoV‐2 spike protein content, indicating inhibition of entry (Figure 1D,E).

**FIGURE 1:**
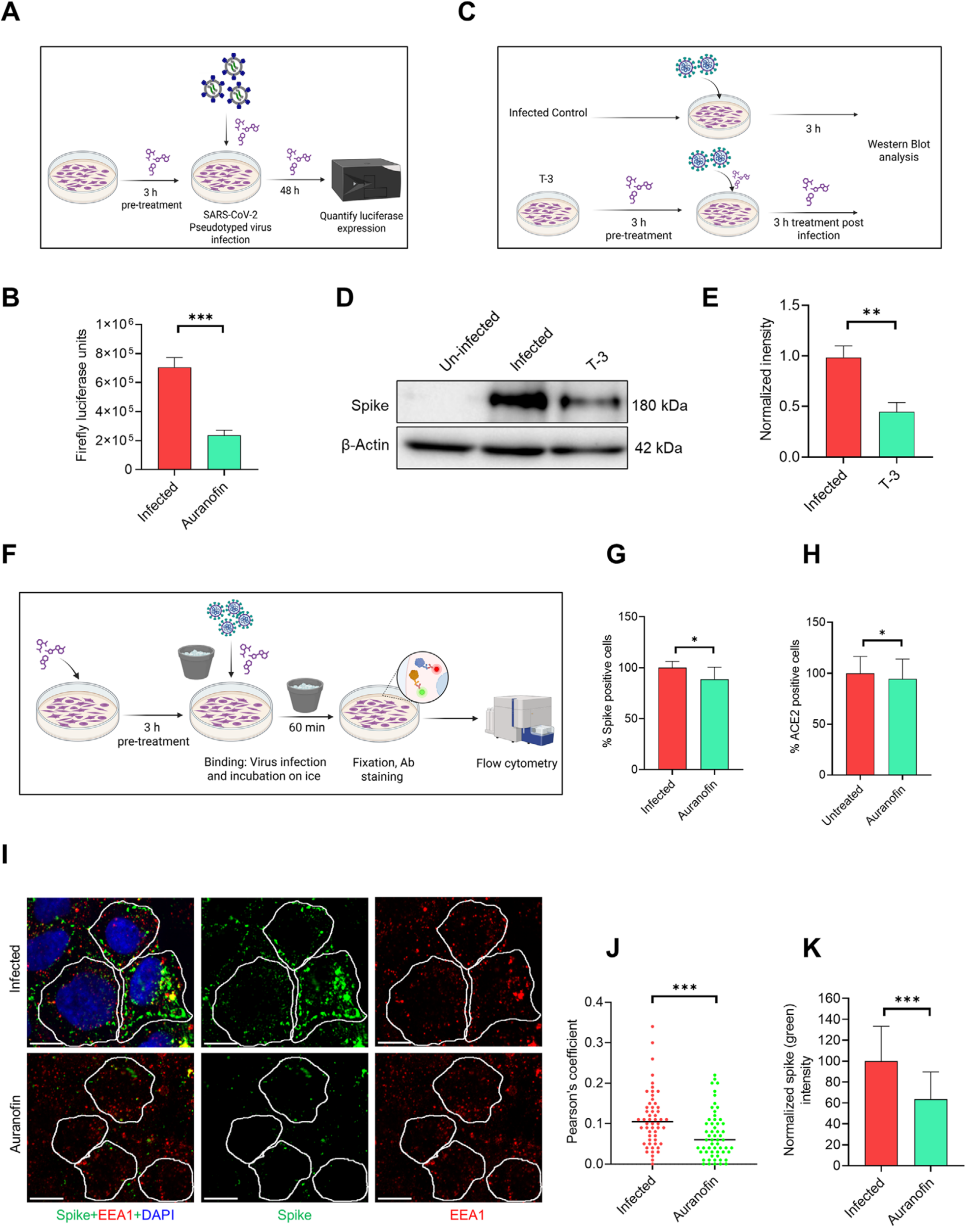
Auranofin restricts SARS‐CoV‐2 entry. (A) Schematic of SARS‐CoV‐2 spike pseudotyped particle entry assay workflow. HEK293‐ACE2 cells were pretreated with 1 µM Auranofin for 3 h and infected with firefly luciferase‐expressing SARS‐CoV‐2 spike pseudotyped particles. After 3 h, cells were lysed, and luciferase activity was measured using a multimode plate reader. (B) Shows firefly luciferase activity for infected control and Auranofin‐treated conditions. (C) Schematic of time‐of‐addition assay workflow for testing the effect of Auranofin on SARS‐CoV‐2 entry. HEK293‐ACE2 cells pretreated for 3 h with 1 µM Auranofin were infected with 10 MOI SARS‐CoV‐2 in the presence of drug (T‐3). After 3 h of incubation, the expression of SARS‐CoV‐2 spike was analyzed by western blot analysis, as shown in (D). The relative density of spike protein levels normalized to the loading control is shown in (E). (F, G) Schematic representation of the virus binding assay workflow. HEK293‐ACE2 cells were pre‐treated with 1 µM Auranofin and incubated with 50 MOI SARS‐CoV‐2 on ice for 1 h in the presence of the drug. Cells were then washed, fixed with 4% paraformaldehyde, and stained for SARS‐CoV‐2 spike. The total number of spike‐positive cells was quantified by flow cytometry, shown in (G). (H) HEK293‐ACE2 cells were treated with 1 µM Auranofin for 3 h, trypsinised and fixed with 4% PFA. Cells were stained for ACE2 and analyzed by flow cytometry. The graph shows the percentage of ACE2‐positive cells compared to the untreated control. (I–K) HEK293‐ACE2 cells pre‐treated with 1 µM auranofin were infected with 10 MOI SARS‐CoV‐2 on ice for 1 h and moved to 37°C in the presence of the drug for 20 min. (I) Confocal microscopy images with virus spike and EEA‐1‐positive early endosomes are shown in green and red, respectively. Individual cells are indicated by ROIs drawn along with cell boundaries using bright‐field images as a reference. (J) Colocalization between spike and EEA‐1 positive vesicles was quantified using ImageJ/Fiji, and Pearson's coefficients are plotted from an average of 10 ROIs per image field. Fluorescence intensity of spike‐positive vesicles quantified from the green channel is shown in (K). Data in panels (B), (J), and (K) are representative of two independent experiments. All other data are representative of three independent experiments. ^∗^ *p* < 0.05, ^∗∗^ *p* < 0.01, ^∗∗∗^ *p* < 0.001; using two‐tailed unpaired *t*‐test. Error bars represent mean ± SD.

Additionally, we checked the effect of drug pretreatment on ACE2 expression on the cell surface and virus attachment by a FACS‐based assay and observed very minimal impact on either (Figure 1F–H). Confocal microscopy analysis showed relatively continuous ACE2 staining, which was enriched at the cell periphery and consistent with surface‐associated receptor distribution (Figure S1A). In contrast, Auranofin‐treated cells displayed more diffuse and heterogeneous ACE2 distribution (Figure S1B). Indeed, a previous study has reported the effect of Auranofin on increasing the mobility of the ACE2 receptor in the plasma membrane [31]. While these changes in ACE2 distribution on the plasma membrane may contribute to the reduced SARS‐CoV‐2 entry phenotype [34] observed in our study, we cannot exclude the possibility that they instead reflect a broader effect of Auranofin on host cell membrane organization or signaling [31].

Our subsequent sets of experiments were then focused on trying to understand the possible mechanisms by which Auranofin inhibits SARS‐CoV‐2 entry. Towards this, we first studied the direct interaction between Auranofin and the SARS‐CoV‐2 spike RBD using Isothermal Titration Calorimetry (ITC). SDS‐PAGE analysis confirmed the purity of the SARS‐CoV‐2 spike RBD protein used for ITC experiments (Figure S2A), and ITC results indicated very weak affinity between Auranofin and spike RBD (K_D_ = 1135 ± 84 µM) (Figure S2B). Next, we examined whether Auranofin affects spike structural integrity or oligomerization by incubating the drug with cells that were either transfected to express SARS‐CoV‐2 spike protein or infected with live virus. Non‐reducing PAGE analysis of virus spike in both cases showed the protein migrated as a single species corresponding to its expected molecular weight, with no additional bands or mobility shifts detected (Sup Figure S2C,D). Therefore, the observed effects of Auranofin on SARS‐CoV‐2 virus entry are independent of gross structural destabilization in the spike oligomeric state or native conformation following de novo synthesis or during infection. Since spike proteolytic maturation is essential for efficient membrane fusion, we next examined whether Auranofin alters the intracellular trafficking of SARS‐CoV‐2 particles upon entry into cells. To this end, we performed virus entry assays in HEK293‐ACE2 cells and analysed drug‐treated cells by confocal microscopy. Auranofin treatment resulted in a significant reduction in colocalization between SARS‐CoV‐2 spike protein and EEA1‐labelled early endosomes (Figure 1I,J), indicating impaired trafficking of incoming viral particles to early endosomal compartments. Consistent with this, spike‐associated fluorescence intensity was significantly reduced upon treatment with 1 µM Auranofin (Figure 1K), suggesting decreased viral internalisation and/or altered post‐entry trafficking. Uninfected cells treated with Auranofin showed a dispersed phenotype of EEA‐1‐positive endosomes throughout the cytoplasm, compared to a predominantly perinuclear localisation in the control (Figure S3A,B). Collectively, these findings indicate that Auranofin‐mediated inhibition of SARS‐CoV‐2 entry is unlikely to result from impaired engagement of the spike receptor‐binding domain or virus attachment to the cell surface. Instead, Auranofin appears to interfere with multiple host‐dependent processes required for efficient entry, including altered ACE2 spatial localization and membrane mobility, and intracellular trafficking of incoming viral particles.

### 2.2 Auranofin Inhibits SARS‐CoV‐2 Mpro Activity but not RdRp‐Dependent Viral Replication

After viral entry, the next step is translation of the incoming viral genome into a polyprotein, followed by processing by the viral proteases Mpro (or 3CLpro; also called the main protease) and PLpro (papain‐like protease) [35, 36]. To evaluate Auranofin's impact on SARS‐CoV‐2 main protease (Mpro) activity, we employed the previously established FlipGFP‐Mpro assay [37]. This system uses a GFP‐based reporter to monitor viral protease activity by exploiting the ability of SARS‐CoV‐2 Mpro, encoded by NSP5, to cleave a specific substrate, thereby triggering reassembly of split‐GFP fragments and producing a measurable increase in fluorescence [37]. HEK293 cells were co‐transfected with the FlipGFPMpro reporter and NSP5 WT (wild type) expression plasmids and 3 h later cell were exposed to 1 µM Auranofin or 7.8 µM Nirmatrelvir, a known SARS‐CoV‐2 Mpro inhibitor (Figure 2A). After 36 h, a ∼40% reduction in GFP intensity was seen in the presence of Auranofin, suggesting that Auranofin significantly inhibits SARS‐CoV‐2 Mpro activity (Figure 2B).

**FIGURE 2:**
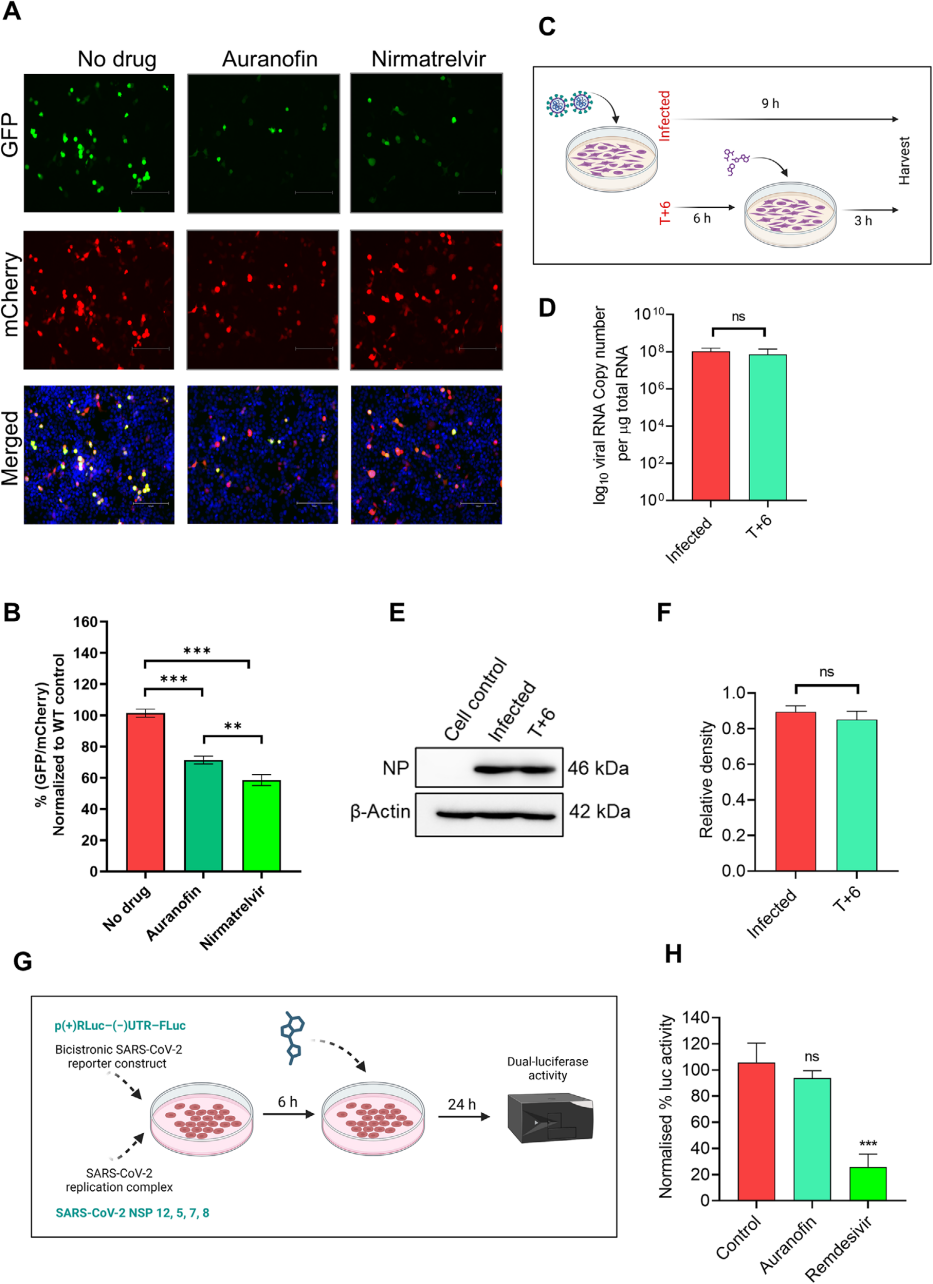
Auranofin inhibits SARS‐CoV‐2 main protease but doesn't affect viral polymerase activity. (A, B) HEK293 cells were co‐transfected with plasmids expressing the FlipGFP‐Mpro reporter construct and SARS‐CoV‐2 Mpro, and 1 µM Auranofin was added after 3 h. After 36 h, cells were fixed with 4% PFA, and the expression of GFP and mCherry was visualised by fluorescence microscopy. The flipGFP fluorescence serves as a readout of Mpro protease activity, whereas mCherry acts as a transfection and expression control. Treatment with Nirmatrelvir served as a positive control to inhibit SARS‐CoV‐2 Mpro activity. Representative fluorescence images showing differences in GFP fluorescence intensity in 3 different conditions are shown in (A). Total GFP and mCherry signals from each field were quantified using ImageJ/Fiji; GFP signals were normalized to mCherry and subsequently normalized to the no‐drug control, as shown in (B). (C) Schematic of time‐of‐addition assay workflow investigating late events of the SARS‐CoV‐2 life cycle. HEK293‐ACE2 cells were infected with 10 MOI SARS‐CoV‐2 and 6 hpi, treated with 1 µM Auranofin (T+6) for 3 h before harvesting samples in RNAIso or Laemmli buffer for (D) qRT‐PCR of SARS‐CoV‐2 vRNA and (E) western blot analysis of virus NP levels, respectively. Relative density of NP protein levels is shown in (F). (G) Schematic for a cell‐based SARS‐CoV‐2 RdRp reporter assay. HEK293 cells were co‐transfected with a bicistronic SARS‐CoV‐2 reporter containing positive‐sense RLuc and negative‐sense Fluc, and an NSP12 plasmid expressing SARS‐CoV‐2 RdRp along with accessory proteins NSP 5, 7, and 8. Transfected cells were then treated with either 1 µM Auranofin or 10 µM Remdesivir after 6 h and harvested after 24 h for quantification of firefly and Renilla dual‐luciferase activity, as shown in (H). Data in panels (D), (E), and (F) are representative of two independent experiments. All other data are representative of three independent experiments. ^∗^ *p* < 0.05, ^∗∗^ *p* < 0.01, ^∗∗∗^ *p* < 0.001; ns, using two‐tailed unpaired *t*‐test. Error bars represent mean ± SD.

Following viral genome translation and polyprotein processing, the viral polymerase assembles to drive genome transcription and replication [38]. To determine whether Auranofin interferes with these post‐entry processes, we employed a time‐of‐addition strategy, treating HEK293‐ACE2 cells with Auranofin at 6 h post‐infection (hpi) (Figure 2C). Viral RNA (vRNA) levels were unaffected by this treatment (Figure 2D), indicating that Auranofin does not suppress viral transcription. Consistent with this, SARS‐CoV‐2 nucleoprotein (NP) expression remained unchanged (Figure 2E,F), suggesting that viral protein translation is equally unimpaired. To further substantiate these findings, we assessed the effect of Auranofin on SARS‐CoV‐2 RNA‐dependent RNA polymerase (RdRp) activity using a previously established replicon‐based dual‐luciferase reporter assay [39]. In this system, cells were co‐transfected with a bicistronic reporter construct, p(+)RLuc‐(−)UTR–FLuc, alongside a SARS‐CoV‐2 RdRp‐expressing plasmid. The construct drives constitutive Renilla luciferase expression as a host‐controlled internal reference, while firefly luciferase expression depends strictly on RdRp‐mediated conversion of a negative‐sense viral RNA template into translatable positive‐sense RNA [39], thereby providing a selective readout of RdRp activity (Figure 2G). Auranofin or Remdesivir was added at 6 h post‐transfection, and luciferase activity was measured at 24 h. Unlike Remdesivir, Auranofin produced no significant reduction in RdRp‐driven firefly luciferase signal (Figure 2H), confirming that it does not interfere with RdRp function. Together, these data demonstrate that Auranofin inhibits SARS‐CoV‐2 Mpro activity without disrupting viral RNA synthesis, protein expression, or RdRp function, suggesting its antiviral mechanism operates largely independent of post‐entry replication suppression. Importantly, the mechanistic distinction between Auranofin and Remdesivir, which acts as a direct RdRp inhibitor, provides a compelling rationale for exploring their combination as a potential antiviral strategy, in which the two agents may target different stages of the viral life cycle in a complementary and potentially synergistic manner.

### 2.3 Auranofin Directly Associates With SARS‐CoV‐2 Mpro and Competes With Nirmatrelvir for Substrate Binding

To characterize the direct interaction of Auranofin with SARS‐CoV‐2 Mpro, we combined computational docking with biophysical binding analysis. Guided structural docking [40] revealed that Auranofin occupies a shallow pose within the Mpro catalytic cleft, proximal to the His41–Cys145 catalytic dyad (Figure 3A), in contrast to Nirmatrelvir, which engages the substrate‐binding pocket in a deeper binding mode with extensive contacts across key residues including His41, Cys145, Gly143, and Glu166 (Figure S4A). To validate direct physical binding, we performed ITC using purified Mpro, whose purity and molecular weight were confirmed by SDS‐PAGE (Figure 3B). Auranofin bound Mpro with a dissociation constant (K_D_) of 197 ± 16.6 µM (Figure 3C), approximately eight‐fold weaker than Nirmatrelvir (K_D_ = 25 ± 2.5 µM; Figure S4B), consistent with Nirmatrelvir's superior binding free energy of −9.72 kcal/mol, roughly 1.4‐fold lower than that of Auranofin (Figure S4C). Notably, binding affinity for the catalytic mutant C145A was markedly reduced (K_D_ = 555 ± 127 µM; Figure 3D), approximately three‐fold higher than for wild‐type Mpro, implicating the active site as the primary binding locus for both compounds. Sequential binding experiments further revealed mutual competition between the two ligands. Auranofin showed greatly diminished binding to the Mpro‐Nirmatrelvir complex (K_D_ ≈ 909 ± 34 µM; Figure 3E), while Nirmatrelvir partially displaced Auranofin from the Mpro‐Auranofin complex (K_D_ ≈ 102 ± 4.8 µM; Figure S4D), collectively indicating overlapping binding sites and competitive interference. Although ITC confirmed that both compounds bind Mpro spontaneously with thermodynamically favorable parameters, their mutual competition suggests that combining Auranofin with Nirmatrelvir would be counterproductive, as co‐administration is likely to attenuate rather than enhance Mpro inhibition.

**FIGURE 3:**
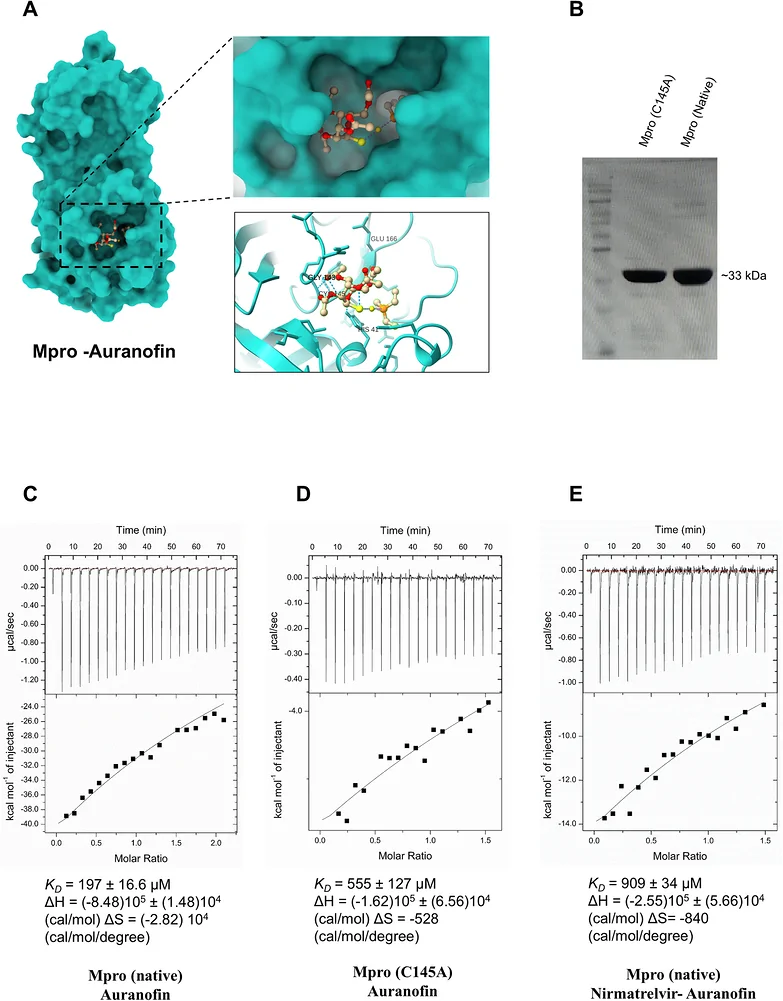
Auranofin partially binds to SARS‐CoV‐2 Mpro to inhibit its activity. Binding free energy and docking of Auranofin against SARS‐CoV‐2 Mpro were performed using AutoDock. (A) Docked structures of SARS‐CoV‐2 Mpro with Auranofin and corresponding hydrogen‐bonding interactions with key amino acids. (B) SDS‐PAGE analysis of SARS‐CoV‐2 Mpro and Mpro (C145A) used for the above experiments. (C) ITC Sensogram and isotherm showing binding interactions between Mpro and Auranofin. (D, E) Similar ITC dataset for binding interactions between Auranofin and the C145A catalytic mutant of Mpro is shown in (D). (E) Shows ITC sensogram and isotherm parameters for Auranofin binding to the preincubated native Mpro‐Nirmatrelvir complex.

### 2.4 Auranofin Abrogates SARS‐CoV‐2 Spike‐Mediated Syncytia Formation and Inhibits Spike Protein Processing

Syncytia formation is a hallmark late‐stage consequence of SARS‐CoV‐2 infection in permissive cells, arising from spike protein‐mediated cell‐cell fusion that produces multinucleated giant cells [41, 42]. To evaluate the effect of Auranofin on this process, we employed a split‐GFP complementation assay in which GFP 1–10 and GFP 11 fragments are expressed in separate cell populations, generating a fluorescence signal exclusively upon cell fusion [42] (Figure 4A). HEK293 cells co‐expressing SARS‐CoV‐2 spike and GFP 1–10, pre‐treated with 1 µM Auranofin, were co‐cultured with HEK293‐ACE2 cells expressing GFP 11 in the continued presence of the drug. GFP fluorescence analysis at 12 h revealed an approximately 50% reduction in spike‐induced syncytia formation (Figure 4B,C), indicating that Auranofin substantially impairs virus spike‐driven cell‐cell fusion. Flow cytometry analysis demonstrated an approximately 40% reduction in the proportion of spike‐positive cells, accompanied by a corresponding decrease in surface spike expression as reflected by reduced median fluorescence intensity (MFI) (Figure 4D,E). Since spike protein interaction with ACE2 and its proteolytic cleavage into S1/S2 fragments are prerequisites for both syncytia formation and viral entry [43], we next examined whether Auranofin influences spike processing.

**FIGURE 4:**
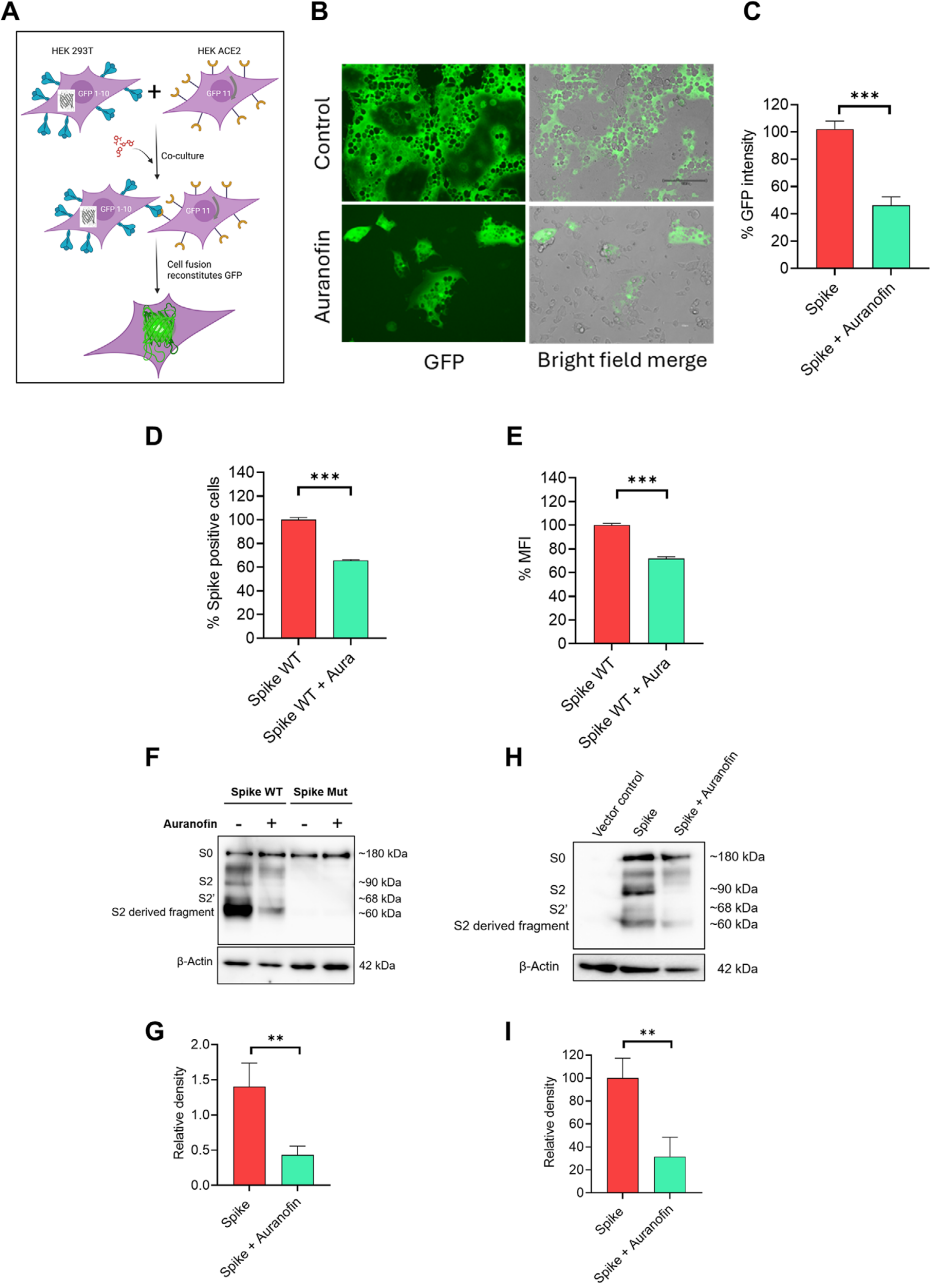
Auranofin inhibits SARS‐CoV‐2 spike‐mediated syncytia formation and spike processing. (A) Schematic showing the split‐GFP‐based reporter system for studying the cell‐cell fusion assay. (B) HEK293 cells expressing SARS‐CoV‐2 spike and GFP 1–10 were pre‐treated with 1 µM Auranofin and co‐cultured with HEK293‐ACE2 cells expressing GFP 11 in the presence of the drug. After 12 h, cells were fixed with 4% PFA, and images were captured using a fluorescence microscope. Representative images show fused cells expressing GFP as a marker for SARS‐CoV‐2 syncytia formation. (C) Shows average GFP intensity from different fields quantified using a high‐content screener. (D, E) HEK293 cells expressing SARS‐CoV‐2 spike were co‐cultured with HEK293‐ACE2 cells in the presence of 1 µM auranofin, and 12 h later, they were fixed with 4% PFA and stained for spike and analyzed by flow cytometry. The total number of spike‐positive cells and the median fluorescence intensity are shown in (D) and (E), respectively. (F) HEK293‐ACE2 cells were transfected with plasmids expressing either wild‐type SARS‐CoV‐2 spike or a fusion mutant with a K814R substitution at the S2′ site, and 6 h later treated with 1 µM auranofin. Cell lysates were collected 24 h post‐treatment, and expression of spike protein was analyzed by Western blot. The relative S2/S0 ratio band intensity is shown in (G). (H, I) HEK293 cells expressing SARS‐CoV‐2 spike were co‐cultured with HEK293‐ACE2 cells in the presence of 1 µM auranofin, and 12 h later, cell lysates were used to analyze differences in spike protein cleavage by Western blot, as shown in (H), and the spike cleavage fragments S2 to S0 ratio is shown in (I). All data are representative of three independent experiments. ^∗∗^ *p* < 0.01, ^∗∗∗^ *p* < 0.001; ns, using two‐tailed unpaired *t*‐test. Error bars represent mean ± SD.

Auranofin is a potent inhibitor of the thioredoxin reductase system and has been reported to perturb intracellular redox homeostasis and thiol‐dependent cellular processes [44, 45]. Given that many proteases rely on redox‐sensitive catalytic residues or thiol‐dependent regulation for activity [46], we hypothesized that Auranofin may also affect host proteases involved in SARS‐CoV‐2 entry. Proteolytic processing of spike at the S2′ position by host serine proteases such as TMPRSS2, or by endosomal cathepsins following endocytosis, facilitates exposure of the fusion peptide that aids in membrane fusion and virus entry [47, 48]. Cleavage at the S1/S2 junction is mediated by furin proteases during viral biogenesis within the trans‐Golgi network, which then generates fusion‐competent virions [48, 49]. HEK293‐ACE2 cells were transfected with SARS‐CoV‐2 spike, treated with 1 µM Auranofin 6 hpi, and lysates were harvested 24 h later. Western blot analysis using an antibody directed against the C‐terminal S2 domain (1029–1192 a.a.) detected full‐length spike (S0), a prominent ∼90 kDa band consistent with S2 cleavage product, a faint band at ∼68 kDa consistent with putative S2′ [50], and a lower‐molecular‐weight S2‐reactive fragment migrating at ∼60 kDa, which may represent a downstream S2‐derived processing product (Figure 4F). Moreover, cells expressing a spike fusion mutant with a K814R substitution at the S2′ site markedly reduced the generation of all detectable cleavage products (Figure 4F). These findings support the assignment of the observed ∼68 kDa band as an S2′‐derived cleavage product [50] and in turn suggest that the lower‐molecular‐weight ∼60 kDa fragment is generated through subsequent processing events downstream of S2′ cleavage. Importantly, Auranofin treatment reduced spike S1/S2 processing, as evidenced by a decreased S2/S0 ratio compared with vehicle‐treated cells (Figure 4F,G).

We also examined the effects of Auranofin in a co‐culture model wherein HEK293 cells expressing SARS‐CoV‐2 spike were co‐cultured with HEK293‐ACE2 cells and treated with 1 µM Auranofin immediately after seeding. Western blot analysis of spike cleavage products following 12 h treatment revealed near‐complete abolition of the S2 cleavage fragment, with an approximately 70% decrease in the S2/S0 ratio (Figure 4H,I). While S2 cleavage products were detectable in both spike‐transfected monocultures and co‐cultures (Figure 4F), Auranofin consistently prevented S2 fragment accumulation across both conditions. Notably, co‐cultures exhibited higher baseline levels of the S2 product alongside greater retention of uncleaved S0 compared to spike‐transfected cells alone, suggesting that spike‐ACE2 engagement alters spike processing dynamics, a process effectively disrupted by Auranofin.

Collectively, these findings identify inhibition of spike proteolytic maturation as a likely mechanism underlying Auranofin's anti‐syncytial and antiviral activity. Given that SARS‐CoV‐2 syncytia formation facilitates direct cell‐to‐cell viral transmission [51, 52], Auranofin's capacity to suppress this process represents an additional and mechanistically distinct dimension of its antiviral potential.

### 2.5 Auranofin Attenuates NF‐κB Signaling During SARS‐CoV‐2 Infection

Auranofin is a well‐established anti‐inflammatory agent that suppresses NF‐κB activation and downstream inflammatory gene expression. In line with this, we have previously demonstrated that Auranofin mitigates SARS‐CoV‐2 infection in Syrian golden hamsters while concomitantly suppressing pulmonary IL‐6 production [30], a cytokine whose expression is canonically driven by NF‐κB signaling, suggesting that Auranofin's anti‐inflammatory activity may contribute meaningfully to its antiviral efficacy in vivo. To directly interrogate Auranofin's effect on the NF‐κB pathway, we employed a well‐established dual‐luciferase reporter assay in HEK293 cells co‐transfected with an NF‐κB promoter‐driven firefly luciferase construct, a constitutively active Renilla luciferase plasmid (pRLTK) as an internal control, and an empty vector. Following 24 h of transfection, cells were treated with Auranofin and stimulated with TNF‐α to activate the NF‐κB pathway (Figure S5A). Luciferase activity measured at 12 h revealed approximately 30% inhibition of NF‐κB‐driven transcription in Auranofin‐treated cells (Figure S5B). Importantly, when assessed in the context of active SARS‐CoV‐2 infection, Auranofin produced a more pronounced effect, reducing NF‐κB activation by over three‐fold (Figure S5C), accompanied by an approximately two‐fold decrease in TNF‐α expression (Figure S5D). Taken together, these findings confirm that Auranofin exerts meaningful anti‐inflammatory activity through suppression of the NF‐κB pathway, and that this effect is further amplified during SARS‐CoV‐2 infection. The Auranofin‐mediated reduction of IL‐6 and TNF‐α, both NF‐κB‐driven mediators of hyperinflammation, suggests that its therapeutic benefit during SARS‐CoV‐2 infection extends beyond direct antiviral mechanisms to encompass attenuation of virus‐induced inflammatory pathology.

### 2.6 Auranofin Exhibits Synergistic Antiviral Effects With Nucleoside Analogs in the Cell Culture Model

Next, we examined the potential synergistic or additive effects of Auranofin in combination with DAAs. To this end, we tested Auranofin in combination with widely established SARS‐CoV‐2 DAAs, namely nucleoside analogues Remdesivir [53], Molnupiravir [54], and the SARS‐CoV‐2 Mpro inhibitor Nirmatrelvir [11]. We first estimated the IC90 of the drugs, including Auranofin, using the SARS‐CoV‐2 recombinant infectious clone with the NanoLuc luciferase gene (BEI resources, NR‐54003), hereby referred to as SARS‐CoV‐2 Luc (Figure S6A–D). Next, to experimentally confirm this prediction, synergy was evaluated using the ZIP (Zero Interaction Potency) scoring model across a matrix of limiting‐dose drug combinations. Specifically, we performed synergy studies using IC90, IC45, and IC22.5 values for Molnupiravir and Nirmatrelvir, in combination with IC45 and IC22.5 values for Auranofin. Synergy with Remdesivir was assessed using the drug's IC80, IC40, and IC20 values. Remdesivir alone at IC80 achieved approximately a ∼73% reduction in infection, but when combined with Auranofin at IC45, the reduction increased to ∼86%, which is a ∼13% increase in antiviral efficacy (Figure 5A). A similar increase in effectiveness was observed when cells were treated with both Remdesivir IC40 and Auranofin IC45 (Figure 5A), with a ZIP score of 10.19. Individually, these doses reduced infection by about 64%, whereas their combination reduced infection by 77% (Figure 5A,B). Although similar drug combinations with Molnupiravir and Auranofin also resulted in greater inhibition than individual drugs, the overall synergy was lower (ZIP score 1.96) (Figure 5C,D). Nirmatrelvir and Auranofin combination expectedly showed a negative synergy score of ‐5.89, as both drugs compete for the same target, Mpro (Figure 5E,F). Overall, the Auranofin‐Remdesivir combination exhibited the strongest synergistic antiviral effects (Figure 5B).

**FIGURE 5:**
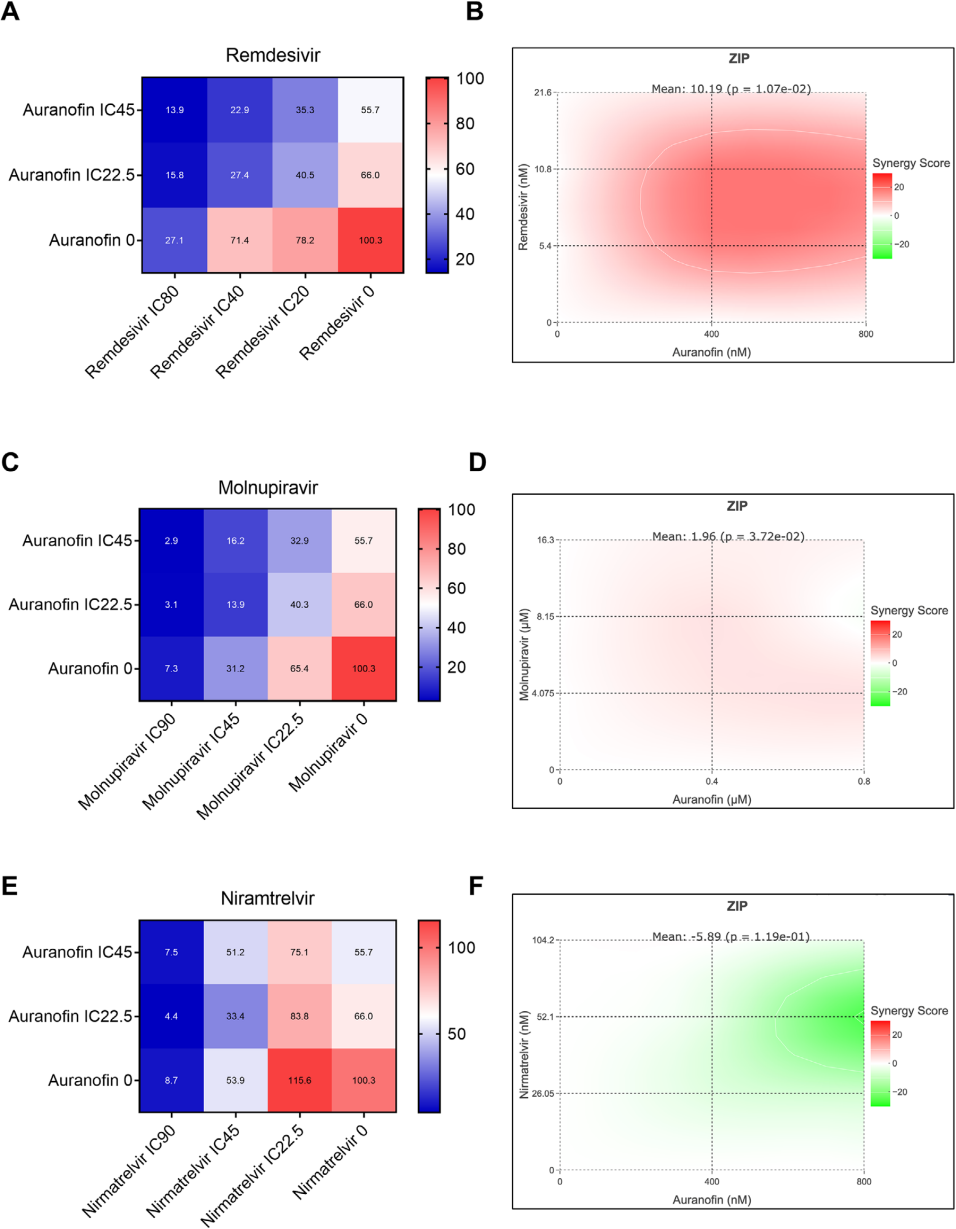
SARS‐CoV‐2 Antiviral efficacy of Auranofin in combination with DAAs in a cell culture model. HEK293‐ACE2 cells were pre‐treated for 3 h with indicated concentrations of Auranofin in combination with Molnupiravir, Nirmatrelvir, or Remdesivir. Cells were then infected with 0.1 MOI SARS‐CoV‐2 Luc in the presence of the above drug combinations, and at 48 hpi, cell lysates were harvested for detection of luciferase activity. Heat maps show the percentage of virus replication, as measured by luciferase reporter activity, for drug combinations of Auranofin with (A) Remdesivir, (C) Molnupiravir, and (E) Nirmatrelvir. (B, D, F) show synergy scores calculated using the Synergy Finder web tool using the Zero Interaction Potency (ZIP) model based on the percentage inhibition values calculated in A, C, and E with GraphPad Prism. A mean ZIP synergy score > 10 was considered synergistic. Auranofin combined with Remdesivir showed synergistic activity (mean ZIP score = 10.19), whereas combinations with Molnupiravir (mean ZIP score = 1.96) and Nirmatrelvir (mean ZIP score = −5.89) did not meet this threshold. The data represent results from 3 independent experiments.

To better recapitulate the synergistic antiviral effects of Remdesivir and, given its exploration in animal models, we employed GS‐621763, an orally bioavailable prodrug of the Remdesivir parent nucleoside, GS‐441524 [55]. We first evaluated the combinatorial activity in vitro using HEK293‐ACE2 cells infected with SARS‐CoV‐2 Hong Kong strain. Co‐treatment with sub‐IC_50_ concentrations of Auranofin and GS‐621763 caused a ~2‐log10 reduction in vRNA load compared to the virus control (Figure 6A). This was > 1‐log magnitude lower than the condition with Auranofin alone, which was also evident by a > 1‐log10 reduction in infectious virus load (Figure 6A,B). A similar trend was seen when the drugs were tested against the Delta variant, albeit at a comparatively reduced magnitude, compared to the SARS‐CoV‐2 Hong Kong strain (Figure 6C). Notably, co‐treatment led to the complete loss of detectable infectious viruses in cell culture supernatants (Figure 6D). The residual RNA signal detected by qRT‐PCR likely reflects the presence of non‐infectious viral genomes or genome fragments that might persist despite the absence of replication‐competent viruses. Upon testing the effect of the drugs against the Omicron variant, Auranofin exhibited only modest antiviral activity on its own, while GS‐621763 alone and combination treatment completely abrogated vRNA and infectious viral loads, which indicates a greater susceptibility of Omicron to the combinatorial treatment (Figure 6E,F).

**FIGURE 6:**
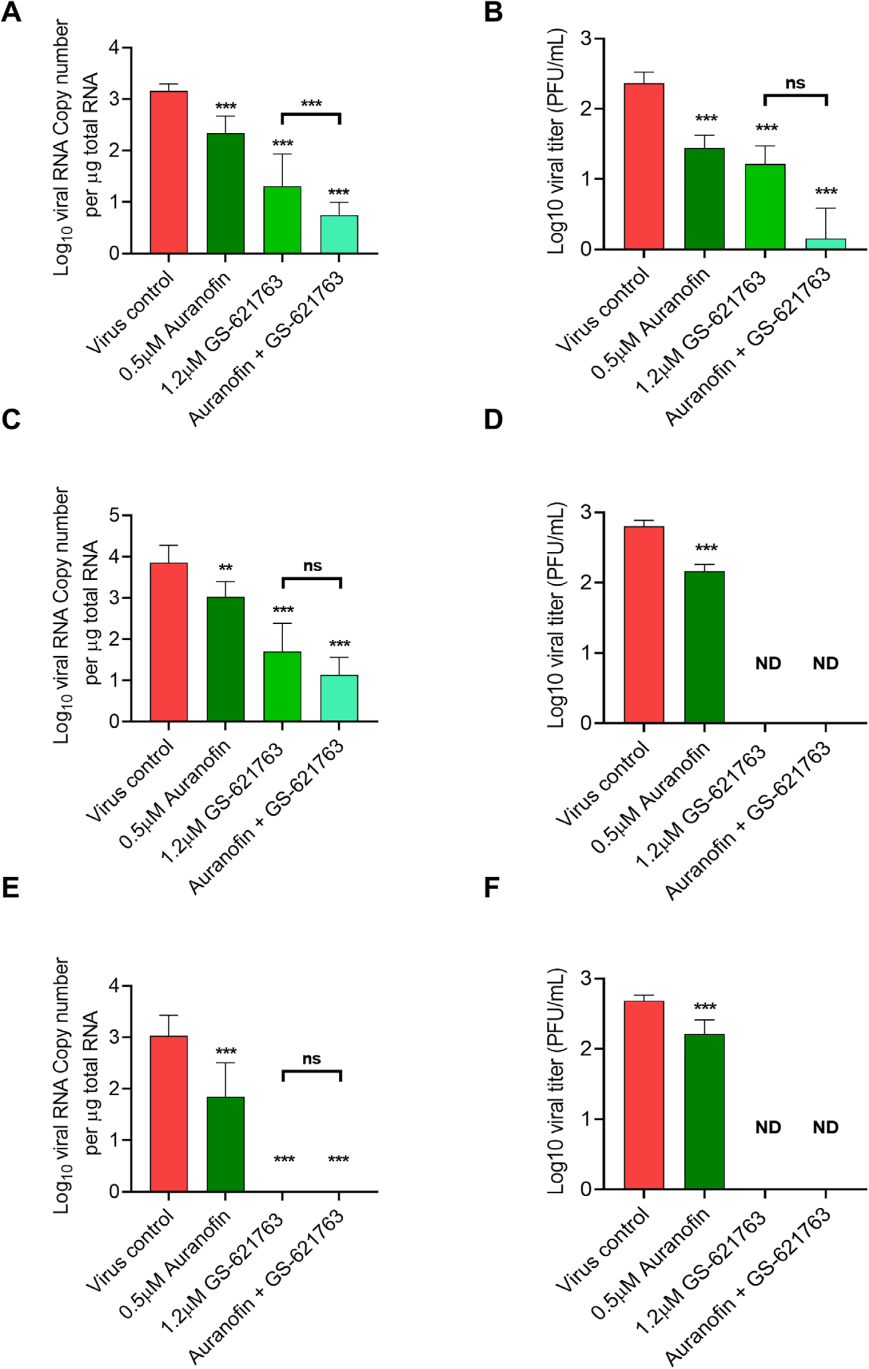
The combination of Auranofin and GS‐621763 shows superior antiviral activity against SARS‐CoV‐2 VOCs. HEK293 ACE2 cells were pretreated with Auranofin and GS‐621763, either individually or in combination, as indicated. Following pretreatment, cells were infected with three different SARS‐CoV‐2 strains at an MOI of 0.01 in the continued presence of the respective drugs. After 48 h, cellular viral RNA levels were quantified by qRT‐PCR. Infectious viral load in supernatants was quantified by plaque assay. (A, B) show quantification data for SARS‐CoV‐2 Hong Kong strain by qRT‐PCR and plaque assay, respectively. Similarly, (C, D) and (E, F) show corresponding qRT‐PCR and plaque assay data for SARS‐CoV‐2 Delta and Omicron variants, respectively. Treated groups were compared with the virus control using one‐way ANOVA, followed by Tukey's multiple comparisons test. Brackets indicate comparisons between GS‐621763 alone and the corresponding GS‐621763 + Auranofin combination. Data represent mean ± SD from three independent biological replicates. ND—Not Detected. ^∗^ *p* < 0.05, ^∗∗^ *p* < 0.01, ^∗∗∗^ *p* < 0.001; ns, non‐significant, using Ordinary one‐way ANOVA with Tukey's multiple comparisons test. Error bars represent mean ± SD.

### 2.7 Auranofin Potentiates the Antiviral Efficacy of GS‐621763 in SARS‐CoV‐2‐Infected K18‐hACE2 Mice

As a follow‐up to the observed synergistic effects in vitro, we tested the combination of Auranofin and GS‐621763 in animal experiments. We first performed pharmacokinetic analyses to determine plasma and lung tissue concentrations of these drugs. GS‐441524, the predominant circulating metabolite of GS‐621763, was quantified in plasma and lung tissue [56]. Auranofin exposure was determined by quantifying total gold (Au) concentrations in plasma and lung tissues [57]. Following a single oral dose of 30 mg/kg body weight (bw) GS‐621763 and 5 mg/kg bw Auranofin combination, GS‐441524 was rapidly detected in plasma, reaching a C_max_ of 5.8 µM at 0.5 h, which declined rapidly thereafter to below the limit of detection (LOD) by 24 h with half‐life (t_1/2_) of 1.93 h (Figure 7A). In lung tissue, GS‐441524 exposure was markedly lower compared to plasma and subsequently declined to below the LOD by 24 h with a half‐life of 3.39 h (Figure 7A). In contrast, gold concentration, as a measure of Auranofin exposure, in plasma and lung tissue were detected at comparatively lower levels at 0.5 h, with Cmax at 4 h, and subsequently plateaued at ∼1 µM at 24 h, well above the LOD (Figure 7B). Based on these observations, a twice‐daily dosing schedule was adopted for subsequent animal experiments to maintain adequate systemic drug exposure. K18‐hACE2 mice, an established preclinical model for SARS‐CoV‐2 infection, were used in this study [58]. Oral administration of the drug combination was well tolerated, with no observable toxicity for up to 5 days (Figure S7A). Experimentally, 30 mg/kg b.w. GS‐621763 and 5 mg/kg b.w. Auranofin were administered bis in die (B.I.D) orally, either individually or in combination. Therapeutic treatment was initiated 12 h post‐infection and continued until day 5 (D5), while prophylactic treatment involved dosing 3 days prior to infection (D‐3) and continued until D5 post‐infection (Figure 7C). Prophylactic treatment groups included two additional groups that received 15 mg/kg b.w. GS‐621763 alone and in combination with 5 mg/kg b.w. Auranofin, respectively, to study the synergistic dose‐sparing effects of these drugs. For infection, animals were intranasally inoculated with 10^5^ PFU of the SARS‐CoV‐2 Delta clinical isolate at D0 and sacrificed at D6 pi (Figure 7C).

**FIGURE 7:**
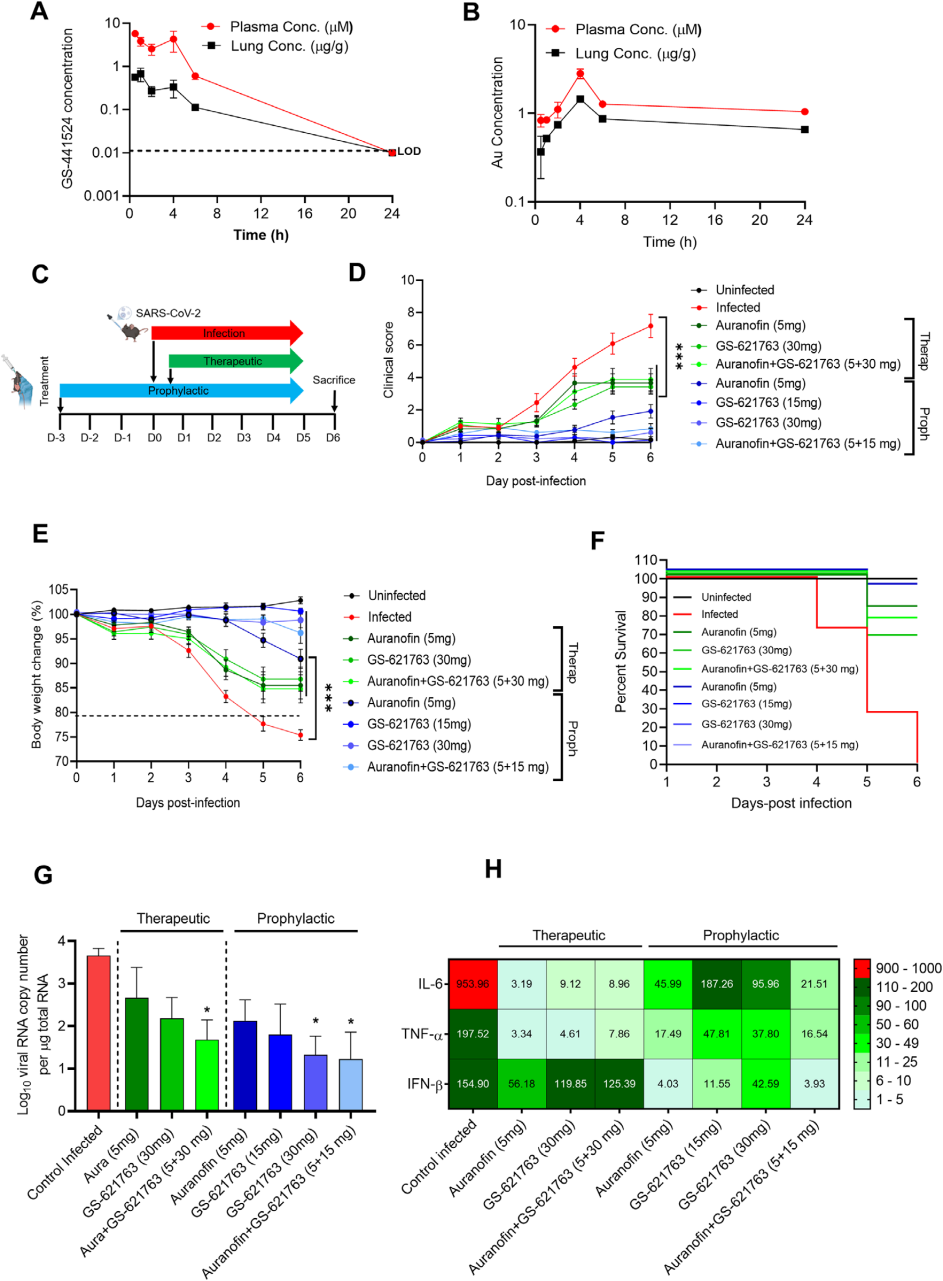
The combination of Auranofin and GS‐621763 provides synergistic protection against SARS‐CoV‐2 infection in the K18‐hACE2 mouse model. (A, B) Pharmacokinetic profiles following a single oral dose of the Auranofin and GS‐621763 combination in C57BL/6 mice. (A) Plasma and lung concentrations of GS‐441524, the active metabolite of GS‐621763. (B) Plasma and lung gold (Au) concentrations as a surrogate marker of Auranofin exposure. (C) Schematic for dosage regimen and infection. Prophylactic treatment was administered from D‐3, and therapeutic treatment was initiated 12 h post‐infection. Animals were infected on D0 and sacrificed at D6 post‐infection. (D) Clinical scores, (E) percentage b.w. loss, (F) survival data, (G) SARS‐CoV‐2 lung vRNA load quantified by qRT‐PCR. (H) Heat map showing expression levels of the pro‐inflammatory cytokines IL‐6 and TNF‐α, and the antiviral cytokine IFN‐β. Bars represent mean ± SEM. Statistical significance was determined using a one‐way ANOVA with a post hoc multiple‐comparison test. Significance is indicated as *(*p* < 0.05). (A, B) represents 1 experiment, *n* = 3 animals per group (C–H) represents 3 independent experiments, *n* = 7–15 animals per group.

SARS‐CoV‐2‐infected control animals exhibited severe clinical symptoms, progressive body weight loss, and complete mortality, consistent with severe disease in this model [59] (Figure 7D–F). This was corroborated by a lung viral RNA load of 3.7 log in this group, which decreased by ∼1.5 log following therapeutic GS‐621763 treatment and by a further ∼1 log in the prophylactic treatment group (Figure 7G). This is consistent with the antiviral activity of GS‐621763, a nucleoside analogue inhibitor of the SARS‐CoV‐2 viral RNA polymerase [54]. Notably, prophylactic administration of GS‐621763 provided greater viral suppression than therapeutic treatment, suggesting that early intervention may enhance antiviral efficacy in this model. Monotherapy with either Auranofin or GS‐621763 partially mitigated disease severity, whereas combination treatment provided the strongest protection, which was reflected by improved b.w. rescue, reduced clinical scores, lung‐to‐body weight ratio, and associated lung pathology across both prophylactic and therapeutic regimens (Figure 7D,E and Figure S7B–D). Although Auranofin monotherapy resulted only in a modest reduction in vRNA load, combination treatments yielded significantly lower levels in both the prophylactic and therapeutic groups (Figure 7G). Moreover, the prophylactic administration of GS‐621763 at 15 mg/kg, in combination with Auranofin, achieved viral reduction comparable to, or greater than, that of GS‐621763 at 30 mg/kg alone. The near‐complete rescue of survival observed in the prophylactic groups (Figure 7F), together with the marked reduction in viral burden (Figure 7G), highlights a dose‐sparing effect and enhanced antiviral efficacy of GS‐621763 when combined with Auranofin. Upon repeated administration of the drug combination B.I.D. for 8 days, GS‐441524 levels were low in plasma and undetectable in lung tissue, whereas Auranofin‐associated gold exposure was sustained in plasma and lung with detectable concentrations (Figure S7E,F). Despite limited detectable lung exposure to GS‐441524, the combination retained antiviral efficacy, suggesting that sustained Auranofin exposure may contribute to the overall therapeutic effect. SARS‐CoV‐2 infection in the control animals led to elevated expression of pro‐inflammatory cytokines, including IL‐6 and TNF‐α, and increased IFN‐β levels, reflecting activation of innate antiviral responses (Figure 7H). All treatment groups showed effective reduction in IL‐6 and TNF‐α levels compared to infected controls (Figure 7H). GS‐621763‐mediated reductions were likely secondary to decreased viral burden, whereas Auranofin monotherapy also attenuated cytokine expression, consistent with its immunomodulatory properties. Although the effect on IFN‐β cytokine levels was limited in the therapeutic group, prophylactic administration of the drugs was much more effective (Figure 7H). Overall, combination therapy in prophylactic groups resulted in the most pronounced reduction in IL‐6 and TNF‐α, along with modulation of IFN‐β, indicating effective suppression of both viral replication and associated inflammatory responses (Figure 7H).

Overall, the all‐oral combination of Auranofin and GS‐621763 outperformed either monotherapy in reducing viral burden, disease severity, and pro‐inflammatory cytokine levels in K18‐hACE2 mice, while Auranofin's dose‐sparing effect enabled a half‐dose of GS‐621763 to achieve comparable or superior viral suppression. These results highlight the therapeutic advantage of pairing Auranofin's immunomodulatory activity with GS‐621763's direct antiviral mechanism in SARS‐CoV‐2 infection.

## 3 Discussion

The COVID‐19 pandemic, the continued emergence of SARS‐CoV‐2 variants [60], and the growing burden of long COVID [61] collectively highlight an urgent and unmet need for therapeutic strategies that simultaneously address direct viral pathology and the dysregulated host inflammatory responses that drive severe disease. Beyond conventional small‐molecule antivirals, metal‐based therapeutics have emerged as a promising class of antiviral agents owing to their ability to interact with nucleophilic cysteine and selenocysteine amino acid residues in viral and host proteins. A distinct advantage of this class of drugs is that they often exert pleiotropic effects through covalent or coordination‐based interactions with multiple proteins [25, 62, 63]. These properties have stimulated considerable interest in repurposing clinically approved metallodrugs for the treatment of COVID‐19. Apart from gold(I) compounds like Auranofin, compounds containing bismuth, ruthenium [30], silver [64], platinum [65], and antimony compounds have been investigated for their ability to inhibit SARS‐CoV‐2 replication by targeting viral proteases, helicases or host pathways required for infection [66]. Despite this breadth of activity, the majority of metallodrugs investigated against SARS‐CoV‐2 have been evaluated as single agents, and comparatively little attention has been given to their combination with established direct‐acting antivirals [67]. This represents an important therapeutic opportunity, as the distinct target profiles of metallodrugs could complement the highly specific inhibition achieved by DAAs, thereby reducing dependence on any single viral target. The FDA‐approved drug Auranofin exemplifies this opportunity, both as a gold(I) compound that retains the pleiotropic, cysteine‐targeting mechanism characteristic of metallodrugs and for its well‐established anti‐inflammatory activity, which provides an additional host‐directed component largely absent from conventional DAAs. Here, we demonstrate that Auranofin exerts antiviral activity against SARS‐CoV‐2 through a multifaceted mechanism that engages multiple stages of the viral lifecycle, from virus entry through Mpro‐dependent proteolysis, spike protein cleavage, and host inflammatory signaling.

Tracing the effects of Auranofin on sequential events during the SARS‐CoV‐2 life cycle, we confirmed the findings of a previous report that Auranofin affects viral entry [31]. Our observation that Auranofin impacts the localization of the ACE2 receptor on the plasma membrane is consistent with this study, which attributed this to the disruption of lipid raft microdomains [31]. This effect could also cause the impaired post‐attachment trafficking of incoming virions that we observed. Notably, the direct assessment of virus attachment revealed only a marginal effect of Auranofin, as did the reduction in post‐attachment endosomal trafficking, which was similarly modest, indicating that these steps are comparatively minor contributors to the overall inhibition of SARS‐CoV‐2 entry. The perturbation of membrane microdomains may have also been linked to the suppression of NF‐κB activation, indicating that altered ACE2 distribution and anti‐inflammatory signalling share a common upstream mechanism [31]. Therefore, ACE2 redistribution upon Auranofin treatment could have multiple ripple effects, ranging from reduced virus entry phenotype [34] to broader antiviral and anti‐inflammatory effects.

Another key DAA target of SARS‐CoV‐2 is Mpro, a cysteine protease responsible for most cleavage events in the viral precursor polyprotein. Mpro mediates proteolytic activity through a catalytic dyad comprising His41 and Cys145, with additional active‐site residues, including Glu166, His163, and Gly143, playing critical roles in substrate binding and catalysis [68, 69]. Notably, Mpro structure and function are highly conserved across coronaviruses, and no human homologue exists, making it an attractive and selective antiviral target with a favorable therapeutic window [70]. Nirmatrelvir, the first protease inhibitor developed specifically against SARS‐CoV‐2 Mpro, exploits this vulnerability and is currently administered in combination with ritonavir as a standard‐of‐care treatment for COVID‐19 [71, 72]. Our identification of SARS‐CoV‐2 Mpro as a direct Auranofin target, supported by computational docking and competitive binding with Nirmatrelvir, adds a virus‐directed mechanism to Auranofin's antiviral profile, complementing its established host‐directed, anti‐inflammatory activity.

Examination of virus lifecycle events occurring between viral protein translation and the start of a new infection cycle revealed a significant reduction in SARS‐CoV‐2 spike‐induced syncytia formation, with direct implications for the cell‐cell spread of new virus particles [51]. This is biochemically coherent with the near‐complete inhibition of spike S1/S2 cleavage we observed. Interference with furin‐ and cathepsin‐dependent spike cleavage, essential for membrane fusion competence, likely constitutes the upstream mechanism limiting both viral entry and syncytia formation.

While testing Auranofin's anti‐inflammatory activity, the observed NF‐κB inhibition has generally been demonstrated at concentrations exceeding the ∼0.5 µM steady‐state plasma level achieved in patients on standard clinical dosing. The concentrations used in our in vitro NF‐κB assays should therefore be interpreted with this caveat in mind; however, we note that the plasma gold concentration achieved in our in vivo model (∼0.62 µg/mL; see below) fell within the human therapeutic steady‐state range, supporting the physiological relevance of the anti‐inflammatory effects observed in vivo. Overall, the antiviral profile of Auranofin, encompassing inhibition of viral entry, spike processing, syncytia formation, and Mpro activity alongside host‐directed NF‐κB suppression, established a compelling rationale for its combination with nucleoside analogue antivirals that act orthogonally on RdRp. Consistent with this, the Auranofin‐Remdesivir combination yielded the highest synergy score among all DAA combinations tested in vitro, surpassing those with Molnupiravir or Nirmatrelvir. Crucially, this synergy was preserved with GS‐621763 across multiple SARS‐CoV‐2 variants, supporting broad clinical relevance. Co‐administration of Auranofin with GS‐621763 significantly enhanced in vivo antiviral efficacy relative to either agent alone in the K18‐hACE2 mouse model, with combination therapy permitting a dose‐reduction of GS‐621763 while matching or exceeding the efficacy of monotherapy. This dose‐sparing effect has potential implications for tolerability in prolonged use. Although prophylactically treated animals receiving 5 mg/kg Auranofin plus 15 mg/kg GS‐621763 exhibited a lower reduction in percent body weight than those receiving 15 mg/kg GS‐621763 alone, this was not statistically significant. Importantly, the combination group showed improved survival and significantly lower lung viral burden. Therefore, the observed difference in percent body weight does not diminish the overall therapeutic benefit of the combination regimen. Although the Auranofin dose used in this study (5 mg/kg) corresponds to a human equivalent dose exceeding the maximum approved clinical dose for rheumatoid arthritis (9 mg/day) [73], pharmacokinetic analysis indicated that the actual systemic exposure achieved was well within established clinical safety margins. Following eight days of B.I.D. oral dosing, plasma gold concentration reached approximately 3.15 µM (∼0.62 µg/mL) (Figure S7F). This measured exposure falls within the steady‐state plasma gold concentration range (0.2–1.0 µg/mL) reported in rheumatoid arthritis patients receiving the maximum approved human dose of Auranofin (9 mg/day) over 12 weeks [74].

The mechanistic basis for this enhanced in vivo efficacy reflects the complementary and non‐overlapping modes of action of the two agents: GS‐621763 suppresses SARS‐CoV‐2 RNA replication via RdRp inhibition, while Auranofin targets host inflammatory pathways, as well as viral entry and Mpro activity. GS‐621763 alone also reduced inflammatory cytokine levels, primarily due to decreased viral load, whereas Auranofin exerted additional, virus‐independent anti‐inflammatory effects. In particular, marked reductions in IL‐6 and TNF‐α were observed with Auranofin treatment, consistent with our in vitro data and with established suppression of NF‐κB signalling downstream of thioredoxin reductase inhibition [31]. Given that IL‐6 and TNF‐α are canonical NF‐κB‐regulated cytokines [75, 76] and central mediators of COVID‐19‐associated immunopathology [77], their suppression likely represents a major contributor to the anti‐inflammatory benefit observed in vivo. The comparatively modest effects of Auranofin on IFN‐β expression are mechanistically expected, as type I interferon induction is governed predominantly by IRF3/IRF7‐dependent signaling rather than NF‐κB [78], rendering it less sensitive to thioredoxin reductase inhibition.

Taken together, our findings indicate that inhibition of the SARS‐CoV‐2 Mpro is the principal antiviral mechanism of auranofin, resulting in disruption of viral polyprotein processing and replication. The observed effects on virus spike protein processing and virus entry appear to constitute an additional, downstream antiviral activity rather than the primary mechanism. In parallel, the established immunomodulatory properties of auranofin may provide a complementary host‐directed effect, particularly during the inflammatory phase of infection. The convergence of these complementary antiviral and host‐directed activities likely contributes to the robust synergy observed with GS‐621763, resulting in enhanced antiviral efficacy, dose sparing, and attenuation of inflammatory pathology in vivo. Collectively, our findings provide preclinical validation for a mechanistically informed, all‐oral Auranofin‐Remdesivir combination strategy for COVID‐19 and highlight host‐directed therapy as a broadly applicable approach for current and future coronavirus threats.

### 3.1 Limitations of the Study

The assays performed to test the effects of Auranofin on SARS‐CoV‐2 spike processing used only HEK293 cells expressing ACE2, which do not express TRMPSS2 in sufficient quantity [79]. Consequently, our observations primarily reflect the impact of Auranofin on the endosomal cathepsin‐mediated entry pathway.

The short‐term Auranofin regimen used in this study achieves systemic drug exposure comparable to levels established as tolerable in long‐term human use, though we acknowledge that direct pharmacokinetic and toxicological data for a short‐course, higher‐dose Auranofin regimen in humans and higher animal models are not yet available. This represents an important consideration for future translational development.

Although Auranofin and GS‐621763 demonstrated strong synergistic inhibition of viral replication in vitro, the magnitude of benefit in vivo was comparatively modest, likely reflecting pharmacokinetic differences that may limit the simultaneous availability of both drugs at the site of infection at optimal concentrations. Co‐delivery platforms enabling sustained, localized release of both agents could be a promising avenue to close this translational gap. While the complementary mechanisms of the two drugs suggest they may together raise the barrier to resistance, this remains to be experimentally validated.

## 4 Methods

### 4.1 Ethics Statement

This study was conducted after approval from the Institutional Human Ethics Committee (Approval Number: IHEC No. 13–11092020), Institutional Bio‐Safety Committee (Approval Number: IBSC/IISc/ST/17/2020) and Institutional Animal Ethics Committee (Approval Number: IAEC/IISc/ST/031/2023), following the Indian Council of Medical Research and Department of Biotechnology recommendations. All experiments involving infectious SARS‐CoV‐2 were conducted in the Viral Biosafety Level‐3 facility at the Indian Institute of Science.

### 4.2 Animal Models

Transgenic K18‐hACE2 mice (initial breeders obtained from The Jackson Laboratory (Strain: 034860, USA)) were maintained and bred in the institutional central animal facility under controlled temperature and humidity, with a 12 h light‐dark cycle, and with ad libitum access to standard feed and water at the Viral Biosafety Level‐3 facility, Indian Institute of Science.

Animals aged 8–10 weeks were used and monitored daily throughout the experiment. An overdose of Ketamine (Bharat Parenterals Limited) and Xylazine (Indian Immunologicals Ltd) was used to sacrifice animals upon completion of the experiment.

### 4.3 Cells and Viruses

The following cell lines were used in this study: HEK293 (NCCS, Pune, India), Vero E6 (CRL‐1586, ATCC). All cell lines were cultured in complete Dulbecco's modified Eagle Medium (12100‐038, Gibco) with 10% HI‐FBS (16140‐071, Gibco), 100 IU/mL Penicillin, and 100 mg/mL Streptomycin (15140122, Gibco) supplemented with GlutaMAX (35050‐061, Gibco). The following SARS‐CoV‐2 isolates were procured from BEI Resources, NIAID, NIH: Isolate Hong Kong/VM20001061/2020, NR52282; NR‐55282, Isolate hCoV‐19/USA/PHC658/2021 (Lineage B.1.617.2; Delta Variant); Isolate hCoV‐19/USA/MD‐HP20874/2021 (Lineage B.1.1.529; Omicron Variant), NR‐56461. SARS‐Related Coronavirus 2, Isolate USAWA1/2020, Recombinant Infectious Clone with Nanoluciferase Gene (icSARS‐CoV‐2‐nLuc), Catalog No. NR‐54003. The SARS‐CoV‐2 clinical isolate used for animal experiments was obtained from a human clinical sample collected at the COVID‐19 diagnostic facility at the Indian Institute of Science, Bengaluru, in accordance with institutional biosafety guidelines. This was done following Institutional Human Ethics Guidelines and informed consent of the COVID‐19 patients. Briefly, viral RNA was extracted from a nasopharyngeal swab sample collected in viral transport medium, using the QIAamp Viral RNA Mini Kit (52906, Qiagen) and sequenced with Oxford Nanopore Technologies using the MinION platform. Basecalling, adapter trimming, and demultiplexing were performed using Guppy. Reads were trimmed by 25 bp from both ends, followed by primer trimming using BBDuk (v38.37), and filtered to retain fragments between 100 and 600 bp. Reference‐based genome assembly was carried out using Minimap2 (v2.17) with increased sensitivity against the SARS‐CoV‐2 reference genome NC_045512. Consensus genome sequences were generated using a 0% majority rule with a minimum coverage depth threshold of 10×, resulting in an average sequencing depth of approximately 1000×. Lineage assignment using Pangolin classified the isolate as B.1.617.2 (Delta). The consensus genome sequence has been deposited in the Indian Biological Data Centre (INDA ID: INGCA000000343) and GISAID (Accession No. EPI_ISL_20293061). All the viruses were propagated and titrated by plaque assay in Vero E6 cells as previously described [80]

### 4.4 Drugs

For animal experiments, Auranofin (15316, Cayman Chemical) was solubilized in 2.5% dimethyl sulfoxide (DMSO) and 97.5% water, whereas GS‐621763 (HY‐145119, MedChem Express) was solubilized in a solvent mixture containing 2.5% DMSO (D2650, Sigma‐Aldrich), 10% Kolliphor (07076, Sigma‐Aldrich), 10% Labrasol (Gattefosse), 2.5% Kollisolv Polypropylene Glycol 400 (06855, Sigma‐Aldrich), 75% water. For cell culture experiments, the drugs were dissolved in DMSO.

### 4.5 Virus Entry Assay

HEK‐ACE2 cells were seeded in poly‐L‐lysine pre‐coated 24‐well plates to reach ∼90% confluency after 24 h. To test the effect on viral entry, cells were pre‐treated with 1 µM Auranofin for 3 h, then infected with SARS‐CoV‐2 at 10 MOI in the presence of the drug. Cells were not washed to remove unbound virus or residual drug. After 3 h of incubation, cells were lysed, and the expression of SARS‐CoV‐2 spike was analyzed by western blotting. Auranofin was present in the media throughout the experiment. Quantification of protein band intensities was done using ImageJ/Fiji.

### 4.6 SARS‐CoV‐2 Cell‐Attachment Assay

For the SARS‐CoV‐2 binding assay, HEK293‐ACE2 cells were trypsinised to form a cell suspension, then resuspended in complete media. Cells were then infected with 50 MOI SARS‐CoV‐2 for 45 min on ice in the presence of 1 µM Auranofin. Unbound virus particles from the cell surface were removed by washing twice with ice‐cold PBS, and cells were fixed using 4% PFA for 10 min. Cells were then washed with PBS and incubated in blocking buffer (PBS containing 2% BSA) for 30 min. Detection of cell‐surface‐bound SARS‐CoV‐2 spike was performed by incubating the cell suspension with a mouse anti‐Spike primary antibody (GTX632604, Genetex) diluted in blocking buffer for 30 min. Cells were then washed with PBS and resuspended in blocking buffer containing anti‐mouse Alexa 488 secondary antibody (A‐11001, Invitrogen) for 30 min. After further washing, the cell population positive for spike surface staining was analysed using a Beckman Coulter CytoFLEX flow cytometer. Data were analysed using CytExpert 2.5.0.77 software with unstained and single‐stained controls.

### 4.7 SARS‐CoV‐2 Mini‐Replicon Assay

The protocol for the SARS‐CoV‐2 replicon assay was as published before [39], with some modifications. Briefly, HEK293‐ACE2 cells in a 24‐well dish were co‐transfected with 250 ng each of SARS‐CoV‐2 F‐luc and RdRp plasmids (kind gift from Subhash C Verma) [39], and after 6 h, the media were replaced with media containing 1 µM Auranofin. 10 µM Remdesivir was used as a positive control. In both cases, cell lysates were collected after 24 h using passive lysis buffer, and firefly/renilla luciferase expression was quantified using the Dual‐Luciferase Reporter Assay System (Promega, E1980) as per the manufacturer's instructions. Luminescence readings were taken using a Varioskan LUX multimode plate reader (Thermo Fisher).

### 4.8 SARS‐CoV‐2 FlipGFP‐Mpro Reporter Assay

HEK293 cells were seeded in poly‐L‐lysine‐coated 24‐well plates and used 24 h later for the experiment. To assess the effect of Auranofin on SARS‐CoV‐2 Mpro activity, HEK293 cells were transfected with 125 ng of FlipGFP‐Mpro plasmid (Addgene #163078) and 375 ng of NSP5 WT plasmid using Lipofectamine 2000 transfection reagent (Invitrogen, 11668019). 3 h post‐transfection, the medium was replaced with fresh DMEM containing 1 µM Auranofin or 7·8 µM Nirmatrelvir. At 36 h post‐transfection, cells were fixed with 4% paraformaldehyde (PFA), and GFP fluorescence was quantified using ImageJ/Fiji.

### 4.9 SARS‐CoV‐2 Transcription Activity Assay

To study the effect of Auranofin on viral polymerase activity, cells were infected with SARS‐CoV‐2 at an MOI of 10. Six hours post‐infection, 1 µM Auranofin was added to the culture medium, and the cells were further incubated for 3 h in the presence of the drug. Cells were then harvested for analysis of SARS‐CoV‐2 nucleoprotein expression by western blotting and quantification of viral RNA (vRNA) copy number by qRT‐PCR.

### 4.10 Spike Cleavage Assay

HEK293‐ACE2 cells seeded in 24‐well plates were transfected with plasmids expressing either wild‐type SARS‐CoV‐2 spike or a spike fusion mutant harboring a K814R substitution at the S2′ cleavage site. 6 h post‐transfection, the culture medium was replaced with fresh medium containing either vehicle (DMSO) or 1 µM Auranofin, and cells were incubated for an additional 24 h. Cell lysates were collected in Laemmli sample buffer, and spike protein expression and processing were analyzed by western blotting using an anti‐SARS‐CoV‐2 spike antibody (GeneTex, GTX632604). The relative S2/S0 band intensity ratio was determined by densitometric analysis using ImageJ/Fiji.

### 4.11 Co‐Culture Assay for Syncytia Formation

To evaluate the effect of Auranofin on SARS‐CoV‐2‐induced syncytium formation by imaging, HEK293 cells seeded in 24‐well plates were co‐transfected with 0·25 µg each of plasmids expressing SARS‐CoV‐2 spike and GFP 1–10. In parallel, HEK293‐ACE2 cells were co‐transfected with 0·25 µg each of empty vector (pLX304) and GFP11. After 24 h, cells were trypsinized, and the two cell populations were co‐cultured at a 1:1 ratio in the presence of vehicle (DMSO) or 1 µM Auranofin, at a total density of 0·2 × 10^6^ cells per well in a 24‐well plate. After 12 h of co‐culture, cells were fixed with 4% PFA, and images were acquired using an EVOS M5000 fluorescence microscope. The total area of GFP fluorescence, indicative of cell‐cell fusion, was quantified using a Cell Insight CX7 High Content Screener (Thermo Scientific). To assess the effect of Auranofin on SARS‐CoV‐2 spike protein cleavage, a similar experiment was performed without the split‐GFP components (GFP1–10 and GFP11). Cell lysates were collected 12 h after co‐culture and analyzed for SARS‐CoV‐2 spike protein expression and cleavage by western blotting.

### 4.12 Confocal Microscopy

#### 4.12.1 ACE2 Immunofluorescence Staining

HEK293‐ACE2 cells were seeded on coverslips coated with poly‐L‐lysine in a 24‐well plate. After 24 h, cells were treated with vehicle (DMSO) or 1 µM Auranofin and, after 3 h, fixed with 4% PFA. ACE2 staining was done by immunofluorescence assay using ACE2 Recombinant Rabbit Monoclonal Antibody (MA5‐32307, Invitrogen). Secondary staining was done using anti‐rabbit Alexa Fluor 488 antibody (A‐11008, Invitrogen). Nuclei were stained using DAPI (D9542, Sigma‐Aldrich). Images were acquired using a Nikon Eclipse Ti2 confocal microscope under identical imaging settings for all conditions.

#### 4.12.2 SARS‐CoV‐2 Entry and Endosomal Colocalization

Briefly, HEK293‐ACE2 cells were seeded onto poly‐L‐lysine‐coated glass coverslips in 24‐well plates. At approximately 80% confluency, cells were pretreated with 1 µM Auranofin for 3 h at 37°C in a humidified incubator with 5% CO_2_. Cells were pre‐chilled on ice for 5 min before infection, then incubated with a 10 MOI SARS‐CoV‐2 inoculum on ice for 1 h with intermittent agitation. These conditions permit viral attachment to the cell surface while minimising virus internalisation. Unbound virus was subsequently removed by washing with warm PBS, and cells were incubated in complete medium containing 1 µM Auranofin for 20 min. Cells were then washed with warm PBS, fixed with 4% PFA for 10 min, and processed for immunofluorescence staining. Cells were stained with rabbit anti‐EEA1 (C45B10, Cell Signaling Technology) and mouse anti‐SARS‐CoV‐2 Spike (GTX632604, GeneTex) primary antibodies. Alexa Fluor 488‐conjugated goat anti‐mouse IgG (A‐11001, Invitrogen) and Alexa Fluor 568‐conjugated goat anti‐rabbit IgG (A‐11011, Invitrogen) were used as secondary antibodies. Images were acquired using a Nikon Eclipse Ti2 confocal microscope under identical imaging settings for all conditions. Colocalization analysis between EEA1‐positive endosomes and spike‐positive vesicles was performed using the Coloc2 plugin in ImageJ/Fiji, and results were expressed as Pearson's correlation coefficient. For each condition, 10 ROIs were analysed per image field across five independent image fields. Total intracellular spike fluorescence intensity was also measured as a surrogate for viral entry.

#### 4.12.3 NF‐κB Reporter Assay

HEK293 cells were seeded into poly‐L‐lysine‐coated 24‐well plates and allowed to settle for 24 h before transfection. Cells were co‐transfected with 50 ng of IgK‐IFN‐luc plasmid expressing a firefly luciferase gene driven by the NFκB promoter (14886, Addgene), 20 ng of pRL‐TK plasmid expressing constitutively active Renilla luciferase (provided by Prof. Adolfo García‐Sastre (Icahn School of Medicine at Mount Sinai, New York) and described before [81], and 500 ng of empty vector. After 24 h, cells were pre‐treated with 1 µM Auranofin for 3 h and stimulated with 50 ng/well of TNF‐α (300‐01A‐100 UG, PeproTech) for 12 h. Luciferase activity was quantified with a dual‐luciferase kit (E1980, Promega) as per the manufacturer's instructions, using a TECAN Infinite 200‐PRO multiplex reader. To evaluate the effect of Auranofin on the NFκB pathway during viral infection, cells were transfected with reporter plasmids as above, but stimulated by infecting with 0.1 MOI SARS‐CoV‐2 for 48 h. Dual luciferase activity was measured as above. Human TNF‐alpha expression levels in infected cells were quantified by qRT‐PCR using the following primers:

Forward primer: 5' AGTGAAGTGCTGGCAACCAC 3'

Reverse primer: 5' GAGGAAGGCCTAAGGTCCAC 3'.

#### 4.12.4 In Silico Drug Docking Analysis and Structure Visualization

The 3D structure of Auranofin was built and optimized using Avogadro. The Nirmatrelvir structure was obtained from DrugBank, and the SARS‐CoV‐2 Mpro structure (PDB: 8DZ2) was retrieved from the Protein Data Bank. The Mpro structure was processed using AutoDock 4.2.6, which included repairing missing atoms, removing water molecules, adding hydrogen atoms, and assigning Kollman charges. Similarly, the structures of Auranofin and Nirmatrelvir compounds were processed by assigning Gasteiger partial charges. The grid box was centered on the Mpro active site with a spacing of 0.297 Å to ensure complete coverage of the binding region. Docking simulations were performed using 200 genetic algorithm runs, with a population size of 500, 27 million energy evaluations, and 100,000 generations. Individual docking poses were clustered using a root‐mean‐square deviation (RMSD) cutoff of ≤2.0 Å. The cluster with the most members and the lowest binding free energy was selected as the preferred docking pose. The best‐docked poses were visualized and analyzed using ChimeraX, where hydrogen‐bonding interactions between drugs and Mpro residues were identified and examined.

#### 4.12.5 Isothermal Titration Calorimetry for Auranofin and Nirmatrelvir With SARS‐CoV‐2 Mpro

Isothermal titration calorimetry was performed using a MicroCal ITC200 instrument (Malvern, Northampton, MA). Binding isotherms generated from the ITC experiments were analyzed by non‐linear regression using the built‐in binding model in Origin software (MicroCal, Inc.). SARS‐CoV‐2 Mpro was purified via Ni‐NTA affinity chromatography as previously [82]. The purified Mpro was dialyzed at 4°C in buffer A (20 mM HEPES, 50 mM NaCl, pH 7.5) for ITC experiments. Stock solutions of both compounds (10 mM) were prepared in DMSO and stored at −20°C. The final DMSO concentration during experiments was maintained at 2% in both cell and syringe samples to eliminate any heat change caused by DMSO. Before the experiments, both the protein sample and the compounds were degassed to prevent bubble formation. For the ITC experiments, Mpro (20 µM) in buffer A was loaded into the titration cell, and a 200 µM final concentration of the compounds prepared in buffer A was placed in the injection syringe. The final concentration of DMSO used during the experiments was 2%. The titration involved 20 injections: one initial injection of 0.4 µL followed by 19 injections of 2 µL each, with a 220‐second interval between injections. The system temperature was maintained at 25°C, and the reference power was set to 8 µcal/s. After obtaining the binding isotherms for both compounds, sequential binding experiments were performed to examine how one compound influences the binding of the other. For these experiments, Mpro (20 µM) was preincubated with 200 µM Auranofin at 4°C for 2 h and loaded into the titration cell. Nirmatrelvir (200 µM) was titrated against the preincubated Mpro‐Auranofin complex under identical experimental conditions. A reverse experiment, where Auranofin was titrated against the Mpro‐Nirmatrelvir complex, was also conducted. The resulting thermograms were analyzed using the Origin software package to determine binding parameters, including the association constant (Ka = 1/Kd), enthalpy (ΔH), and entropy (ΔS).

#### 4.12.6 SARS‐CoV‐2 Infection Experiments in an Animal Model

Transgenic K18‐hACE2 mice (*n* = 7–15) aged 8–10 weeks were randomly allocated to five groups. For treatment groups, mice were administered orally twice a day with 200 µL of Auranofin (5 mg/kg b.w.) or GS‐621763 (15 mg or 30 mg/kg b.w.) or combination of both Auranofin (5 mg/kg b.w.) and GS‐621763 (15 mg/kg b.w.), beginning 3 days before SARS‐CoV‐2 infection for prophylactic group and starting from 12 h post infection for therapeutic group and continued until five days post‐infection (dpi). Control groups received the corresponding vehicle on the same schedule. On Day 0, mice were anaesthetized with Ketamine (60 mg/kg, Bharat Parenterals Limited) and Xylazine (16 mg/kg, Indian Immunologicals Ltd.) mix and infected with 10^5^ PFU of SARS‐CoV‐2 Delta variant in 50 µL of PBS intranasally. Animals were monitored daily for body weight changes and clinical signs throughout the experiment. Mice were monitored daily for the development of clinical symptoms and scored according to predefined severity criteria. Clinical parameters evaluated included lethargy (0 = absent, 1 = mild, 2 = severe), piloerection (0 = absent, 1 = mild, 2 = moderate, 3 = severe), abdominal respiration (0 = absent, 1 = mild, 2 = severe), and hunched back (0 = absent, 1 = mild, 2 = severe). Body weight loss was also included as a clinical parameter and graded on a three‐point scale based on percentage reduction relative to baseline (1%–5% = 1; 5.1%–10% = 2; 10.1%–15% = 3). All clinical observations were recorded for each experimental group. At 6 dpi, mice were euthanised with an overdose of ketamine, and lung tissues were harvested and weighed. Tissue was homogenized with a FastPrep‐24 Classic bead‐beating grinder (116004500, MP Biomedicals). Total RNA was extracted using RNAiso Plus (9109, TaKaRa) reagent and stored at −80°C until use.

#### 4.12.7 Quantification of Viral RNA by RT‐qPCR

SARS‐CoV‐2 viral RNA levels in lung homogenates were measured by real‐time quantitative reverse transcription PCR (RT‐qPCR) targeting the Nucleocapsid‐1 (N1) gene. The assay was performed using the AgPath‐ID One‐Step RT–qPCR Kit (AM1005, Applied Biosystems) in a 10 µL reaction mixture containing 100 ng of RNA per sample, run in a 384‐well plate format. Comparative threshold (Ct) values were obtained, and viral copy numbers were determined using a standard curve generated from SARS‐CoV‐2 genomic RNA. The primers and probe used are as follows: Nucleocapsid‐1 forward:5′‐GACCCCAAAATCAGCGAAAT‐3′, reverse: 5′‐TCTGGTTACTGCCAGTTGAATCTG‐3′, N1 (6‐FAM/BHQ‐1) 5′‐ACCCCGCATTACGTTTGGTGGACC‐3′.

#### 4.12.8 Quantification of Cytokine mRNA Expression by RT‐qPCR

The mRNA expression levels of TNF‐α, IFN‐β, and IL‐6 were quantified using extracted total RNA, with 18S rRNA serving as the internal reference gene. Complementary DNA (cDNA) was synthesized from 1 µg of RNA using the PrimeScript RT Reagent Kit with gDNA Eraser (RR047A, TaKaRa). RT‐qPCR was performed using 2.5 µL of cDNA, 2.5 µL of gene‐specific primer mix, and 5 µL of SYBR Green PCR Master Mix (Applied Biosystems). The primer sequences used were as follows:

TNF‐α forward: 5′‐AGAAACACAAGATGCTGGGACAGT ‐3′,

reverse: 5′‐CCTTTGCAGAACTCAGGAATGG‐3′;

IFN‐β forward: 5′‐ CAGCTCCAAGAAAGGACGAAC ‐3′,

reverse: 5′‐ GGCAGTGTAACTCTTCTGCAT ‐3′;

IL‐6 forward: 5′‐ CTGCAAGAGACTTCCATCCAG‐3′,

reverse: 5′‐ AGTGGTATAGACAGGTCTGTTGG ‐3′.

Relative gene expression was calculated using the 2^−ΔΔCt^ method with 18S rRNA normalization, and the mean log_2_ fold change for each gene was determined for each experimental group.

#### 4.12.9 Pharmacokinetic Analysis of GS‐621763 and Auranofin

Pharmacokinetic studies were performed in C57BL/6 mice following oral administration of Auranofin (5 mg/kg b.w.) and GS‐621763 (30 mg/kg b.w.) in combination. For single‐dose pharmacokinetic analysis, blood and lung tissues were collected at 0.5, 1, 2, 4, 6, and 24 h post‐treatment. For repeated‐dose analysis, animals received twice‐daily oral dosing until day 8, and at 12 h after the final dose, plasma and lung samples were collected and stored at −80°C until analysis. GS‐621763 exposure was assessed by quantifying GS‐441524, the predominant circulating metabolite, as previously described [56]. Plasma and lung samples were processed using solid‐phase extraction. A mobile phase following an isocratic flow using 90% methanol containing 0.1% formic acid was used for separation by UPLC‐MS/MS using an Xterra MS C18 column (Waters Corporation, USA). Detection was performed in positive electrospray ionization mode using the transition m/z 292.168 > 201.934 for GS‐441524, and verapamil was used as the internal standard. Calibration standards for GS‐441524 (MedKoo Biosciences, USA) ranged from 50–10 000 ng/mL (0.17–34.35 µM), with a lower limit of quantification (LLOQ) of 50 ng/mL. Auranofin exposure was determined by quantifying total gold (Au) concentrations in plasma and lung tissues using inductively coupled plasma mass spectrometry (ICP‐MS) following acid digestion. The ICP‐MS conditions included plasma power of 1550 W, auxiliary gas flow of 0.8 L/min, nebulizer flow of 0.9 L/min, collision gas flow of 4.83 mL/min, and dwell time of 0.05 s. The Gold detection was performed using the transition m/z 197 to 115, with Indium as a standard. Calibration standards of Au (Sigma‐Aldrich, USA) ranged from 0.025‐5 µg/mL (0.037–7.37 µM) with an LLOQ of 25 ƞg/mL. Drug concentrations were converted to micromolar (µM) values, and pharmacokinetic parameters including maximum concentration (C_max_), time to maximum concentration (T_max_), area under the concentration‐time curve (AUC), and terminal elimination half‐life (t_1/2_) were determined using non‐compartmental analysis.

#### 4.12.10 Quantification and Statistical Analysis

All statistical analyses used in this manuscript were performed using GraphPad Prism 8.4.3 (GraphPad Software, USA). Information on the statistical method used is mentioned in the legend section of the respective figures. Error bars indicate SEM, unless otherwise mentioned. In all cases, a p‐value < 0.05 is considered significant. Figure illustrations have been generated using BioRender.com.

## Author Contributions

Conceptualization: S.T. Methodology: O.K., R.N., S.K.N., R.S., A.K., S.K., A.S., S.S., G.R.R., and C.N. Data curation and formal analysis: O.K., R.N., S.K.N., S.S., G.R.R., and C.N. Resources: S.T., R.K.S., S.N., and S. Tom. Validation and visualization: O.K., R.N., and S.K.Y. Writing – review & editing: O.K., R.N., S.T., and S.K.N. Funding acquisition, project administration, and supervision: S.T.

## Conflicts of Interest

ST, RN, and OK are inventors on an Indian patent application for the use of Auranofin alone or in combination with other antivirals for COVID‐19 treatment.

## Data availability

The data that support the findings of this study are available within this article. Further details can be requested from the lead contact, Shashank Tripathi (shashankt@iisc.ac.in).

## Supporting information




**Supporting File**: advs78336‐sup‐0001‐SuppMat.docx.
