# Supplementary material for: An Oral Antiviral Combination Against Severe COVID‐19 Enabled by the Synergistic Mechanisms of Action of Auranofin and Remdesivir
Source: Adv Sci (Weinh). 2026 Oct 22:e78336. Online ahead of print. doi: 10.1002/advs.78336 (PMC13691223; doi:10.1002/advs.78336)
Supplement: Supplementary file 1 [file ADVS-9999-e78336-s001.docx]

**SUPPLEMENTARY MATERIAL**

**An oral antiviral combination against severe COVID-19 enabled by the synergistic mechanisms of action of Auranofin and Remdesivir.**

Oyahida Khatun^1,2†^, Rohan Narayan^1,2†^, Santhosh Kambaiah Nagaraj^1,2†^, Rishad Shiraz^1,2^, Abinaya Kaliappan^1,2^, Sumandeep Kaur^1,2^, Ankur Singh^4^, Siv Sanmugam^3^,Gudepalya Renukaiah Rudramurthy^3^, Chakenahalli Naveenkumar^3^, Shridhar Narayanan^3^, Radha Krishan Shandil^3^, Shailly Tomar^4^, Shashank Tripathi^1,2*^

^1^Emerging Viral Pathogens Laboratory, Infosys Wing, Centre for Infectious Disease Research, Indian Institute of Science, Bengaluru, 560012, India.

^2^Department of Microbiology and Cell Biology, Division of Biological Sciences, Indian Institute of Science, Bengaluru, 560012, India.

^3^Foundation for Neglected Disease Research, KIADB Industrial Area, Doddaballapur, Bengaluru, 561203

^4^Molecular Virology Lab, Department of Bioscience and Bioengineering, Indian Institute of Technology Roorkee, 247667, Uttarakhand, India

^†^These authors contributed equally.

*Corresponding author. Contact: [shashankt@iisc.ac.in](mailto:shashankt@iisc.ac.in)

**Supplementary Fig. 1. Auranofin alters cell-surface localisation of ACE2 in HEK293-ACE2 cells.** (A, B) HEK293-ACE2 cells were treated with vehicle (A) or 1 µM Auranofin (B) for 3 h, fixed with 4% paraformaldehyde, and stained for ACE2 (green) and nuclei (DAPI, blue). Representative confocal microscopy images acquired under identical imaging conditions are shown. Enlarged views of the boxed regions are shown in the corresponding insets. Scale bars - 25 µm. Images are representative of two independent experiments.

**Supplementary Fig 2. Auranofin exhibits weak interaction with SARS-CoV-2 spike RBD, does not induce gross structural alterations in the SARS-CoV-2 spike protein.** (A) SDS PAGE analysis of the receptor-binding domain (RBD) of the spike protein. (B) The binding affinity of Auranofin with SARS-CoV-2 spike RBD was analyzed by Isothermal Titration Calorimetry. Results show binding isotherm and sensogram for the drug-protein interaction. (C) HEK293 cells were transfected with a plasmid expressing SARS-CoV2 spike and 1 µM auranofin was added 6 h post transfection. Cell lysates were collected 48 h post treatment spike protein levels were analyzed by native PAGE. (D) HEK293-ACE2 cells were infected with 0.01 MOI SARS-CoV-2 in the presence of 1 µM auranofin and after 48 h infection, spike protein levels lysates were analyzed by native PAGE. Date are from 2 independent experiments.

e

**Supplementary Fig 3. EEA-1 positive endosome localization in un-infected cells treated with Auranofin.** (A-B) HEK293-ACE2 cells were pre-treated with 1 µM Auranofin, incubated on ice for 1 h and moved to 37ºC in the presence of drug for 20 min. Cells were then fixed and labelled with antibodies against spike and EEA-1. Confocal microscopy images show (A) control un-treated and (B) cells treated with 1 µM Auranofin.

**Supplementary Fig 4. Auranofin shows weaker binding to SARS-CoV-2 Mpro compared to Nirmatrelvir.** Binding free energy and docking of Nirmatrelvir against SARS-CoV-2 Mpro were performed using AutoDock. (A) Docked structures of SARS-CoV-2 Mpro to Nirmatrelvir and corresponding hydrogen bonding interactions with key amino acids. (B) ITC Sensogram and isotherm showing binding interactions between Mpro and Nirmatrelvir. (C) Binding free energy for Auranofin and Nirmatrelvir calculated using AutoDock. (D) Sensogram and isotherm parameters for Nirmatrelvir binding to the preincubated native Mpro-Auranofin complex.

**Supplementary Fig 5. Auranofin inhibits NF-κB activity and TNF-α induction during SARS-CoV-2 infection.** (A) Schematic of dual luciferase assay for the analysis of NFkB activity. HEK293 cells were co-transfected with an NF-κB promoter-driven firefly luciferase reporter plasmid and the pRLTK Renilla luciferase plasmid as an internal control. After 24 h, cells were treated with 1 μM Auranofin for 3h, followed by induction of NF-κB pathway using recombinant TNF-α. Dual-luciferase activity analyzed after 12 h is shown in (B). (C) Similarly, the assay was repeated in HEK293 ACE2 cells transfected with the above plasmids but infected with 0.1 MOI SARS-CoV-2 to induce the NFkB pathway. Infected cells were harvested at 48 hpi and dual-luciferase assay data is shown. In (B,C) luciferase activity is normalized to induced untreated control. TNF-α mRNA levels in infected cells, quantified by qRT PCR is shown in (D). (B) shows data from 3 independent experiments. (C) is from 2 experiments and (D) is from 1 experiment. ∗p < 0.05, ∗∗p < 0.01, ∗∗∗p < 0.001; using two-tailed unpaired t-test or Welch ANOVA with Dunnett’s T3 multiple comparison tests, where applicable. Error bars represent mean ± SD.

**Supplementary Fig 6. Calculation of standalone IC50 of Auranofin and DAAs.** HEK293 ACE2 cells were pre-treated with indicated concentrations of Auranofin, Molnupiravir, Nirmatrelvir, or Remdesivir for 3 h, followed by infection with 0.1 MOI SARS-CoV-2 Luc in the presence of drugs. Renilla luciferase activity was measured at 48 hpi and IC90 was calculated using Imagej/Fiji. Percentage infection compared to untreated control, showing calculated IC90 values for Auranofin, Molnupiravir, Nirmatrelvir, and Remdesivir are shown in A, B, C, and D respectively. Data represents results from 3 independent experiments. Error bars represent mean ± SD.

**Supplementary Fig 7. Auranofin–GS-621763 combination ameliorates SARS-CoV-2-induced lung pathology and demonstrates systemic and lung drug exposure.** (A) K18 hACE2 mice were treated with 5mg/kg b.w. Auranofin and 30 mg/kg b.w. GS-621763 in combination, and animal b.w. were monitored up to 5 days. Data is presented as a percentage of b.w. at D0, from 1 experiment. (B) Lung to b.w. ratio and (C) gross pathology score of lungs of all groups (n=7-15) compared to control untreated. (D) Shows representative lung images from each group (scale bar=3 mm). (B-D) represent 3 independent experiments. (E,F) Pharmacokinetics of C57BL/6 mice (n=3) which received B.I.D oral dosing of drug combination for 8 days. (E) Plasma and lung concentrations of GS-441524, the active metabolite of GS-621763. (F) Plasma and lung gold (Au) concentrations as a surrogate marker of Auranofin exposure. Error bars represent mean ± SEM. * represents the values below the lower limit of detection (LOD). Statistical significance was determined using one-way ANOVA with post hoc multiple comparisons test. Significance is indicated as *(p < 0.05), **(p < 0.01), ***(p < 0.001).

**Supplementary Methods**

**Generation of HEK293-ACE2 stable cell line**

Lentivirus preparation: HEK293 cells in 6-well plates were transfected with 1 μg pLENTI_hACE2_PURO (Addgene #155295), 0.6 μg gag-pol, and 0.4 μg VSV-G plasmids (gag pol and VSV-G were a Kind Gift from prof. Adolfo Garcia-Sastre (ISMMS, NY) PMID: 34556855) per well using Lipofectamine 2000 (Invitrogen, 11668019) according to the manufacturer’s instructions. After 3h, the medium was replaced with fresh complete DMEM, and cells were incubated at 37°C with 5% CO₂. Supernatants were collected at both 48 and 72h, pooled together and passed through a 0.4μm syringe filter, and storage in aliquots at −80°C until use. For transduction, lentiviral supernatant was mixed 1:1 (v/v) with complete DMEM containing 1μg/mL polybrene (Merck, TR-1003-G). HEK293 cells in 6-well plates were transduced with 2 mL of this mixture per well and incubated at 37°C with 5% CO₂ for 24 h. The medium was then replaced with fresh DMEM, and after 12h, cells were incubated with selection medium containing 1 μg/mL puromycin (Gibco, A1113-03) for 48h. Once complete cell death was observed in un-transduced controls, the surviving transduced cells were replenished with fresh DMEM and allowed to recover for 12 h before being trypsinized and transferred to a T75 flask. Stable HEK293-ACE2 cells were subsequently maintained in medium containing 0.1 μg/mL puromycin and used for experiments within 4 passages.

**Spike Pseudotyped virus particle entry assay**

Pseudotyped virus particles expressing the SARS-CoV-2 spike protein were prepared as described previously.^1^ HEK293-ACE2 cells were seeded in poly-L-lysine (P9155-5MG, Sigma-Aldrich) pre-coated 96-well plates and used for the experiments upon reaching 70-80% confluency. Cells were pre-treated with 1 μM Auranofin (A6733, Sigma Aldrich) for 3 h, followed by infection with pseudotyped particles in the presence of 10 μg/ml polybrene (TR-1003-G, Merck). Auranofin was present in the media throughout the experiment. After 24 h, luciferase activity was quantified with a Firefly luciferase assay kit (E4550, Promega) using a TECAN multi-mode plate reader.

**Generation of SARS-CoV-2 spike fusion mutant**

The Mammalian expression construct expressing the Wuhan Spike protein in the pCAGGS backbone was acquired from BEI resources (NR-52310). The gene was subcloned in PMD20-T vector using TA cloning kit from Takara (Mighty TA cloning Cat. # 6028) and the construct was further subjected to SDM using DpnI (Thermo Fisher Scientific ER 1701) at position 814-815, changing the residues from KR to AA. This renders the spike protein unable to be cleaved near the fusion peptide, further abrogating the fusion step. After sequencing verification, the protein was cloned back into pCAGGS backbone. Expression was further confirmed by Western blot, and loss of fusogenicity was confirmed by the Split GFP assay. The primers used for TA cloning and site-directed mutagenesis are listed below.

TA cloning forward primer: CATATCTAGAGCCTCTGCTAACCATGTTCATGCCTTC

TA cloning Reverse primer: CGCGAGCTCGAATTTCAGGTGTAGTGCAGTTTCACGC

Forward primer: CCTAGCAAGCCCAGCGCGGCGAGCTTCATCGAGGAC

Reverse primer: GTCCTCGATGAAGCTCGCCGCGCTGGGCTTGCTAGG

**IC90 calculation of the drugs**

HEK293-ACE2 cells were seeded in poly-L-lysine pre-coated 24-well plates and 24 h later cells were pre-treated for 3 h with increasing concentrations of Molnupiravir (BD01383150, BLD pharmatech) (20, 10, 5, 2.5 and 1.25 μM), Nirmatrelvir (HY-138687, Medchem express) (100, 80, 75, 60, 50, 25, 16, 10 nM) or Remdesivir (30354, Cayman Chemicals) (1 μM, 0.5 μM, 0.25 μM, 125 nM, 100 nM, 60 nM, 20 nM, 16 nM, and 4 nM). Cells were then infected with SARS-CoV-2 Luc at 0.1 MOI. After 48 h, luciferase activity was measured using the Promega E2820 Renilla Luciferase Assay System as per the manufacturer’s instructions. Luminescence measurements were taken using a TECAN Infinite 200-PRO multiplex reader. The percentage infection was calculated and normalised to the untreated control, and IC90 was determined in GraphPad Prism.

**Antiviral testing of Auranofin in combination with DAAs**

HEK293-ACE2 cells were seeded in poly-L-lysine pre-coated 24-well plates and 24 h later cells were pre-treated for 3 h with IC90, IC45, or IC22.5 of Molnupiravir, Nirmatrelvir, Remdesivir with IC45 or IC22.5 of Auranofin. Cells were then infected with 0.1 MOI SARS-CoV-2 Luc in the presence of the above-mentioned drug combinations and after 48 h, luciferase activity was measured using Promega E2820 Renilla Luciferase Assay System as per manufacturer’s instructions. Luminescence measurements were taken using a TECAN Infinite 200-PRO multiplex reader. The synergy scores were determined using the SynergyFinder web tool ^2^ employing the Zero Interaction Potency (ZIP) model ^3^.

**References**

1.	Crawford KHD, Eguia R, Dingens AS, et al. Protocol and Reagents for Pseudotyping Lentiviral Particles with SARS-CoV-2 Spike Protein for Neutralization Assays. *Viruses* 2020; **12**(5).

2.	Ianevski A, He L, Aittokallio T, Tang J. SynergyFinder: a web application for analyzing drug combination dose-response matrix data. *Bioinformatics* 2020; **36**(8): 2645.

3.	Yadav B, Wennerberg K, Aittokallio T, Tang J. Searching for Drug Synergy in Complex Dose-Response Landscapes Using an Interaction Potency Model. *Computational and structural biotechnology journal* 2015; **13**: 504-13.
